# Graphene-Based Scaffolds for Regenerative Medicine

**DOI:** 10.3390/nano11020404

**Published:** 2021-02-05

**Authors:** Pietro Bellet, Matteo Gasparotto, Samuel Pressi, Anna Fortunato, Giorgia Scapin, Miriam Mba, Enzo Menna, Francesco Filippini

**Affiliations:** 1 Department of Biology, University of Padua, 35131 Padua, Italy; pietro.bellet@studenti.unipd.it (P.B.); matteo.gasparotto.1@phd.unipd.it (M.G.); 2 Department of Chemical Sciences, University of Padua & INSTM, 35131 Padua, Italy; samuel.pressi@unipd.it (S.P.); anna.fortunato.1@phd.unipd.it (A.F.); 3 Department of Medicine, Harvard Medical School, Boston, MA 02115, USA

**Keywords:** graphene, graphene oxide, reduced graphene oxide, tissue regeneration, 2D-scaffolds, hydrogels, fibers, stem cell differentiation

## Abstract

Leading-edge regenerative medicine can take advantage of improved knowledge of key roles played, both in stem cell fate determination and in cell growth/differentiation, by mechano-transduction and other physicochemical stimuli from the tissue environment. This prompted advanced nanomaterials research to provide tissue engineers with next-generation scaffolds consisting of smart nanocomposites and/or hydrogels with nanofillers, where balanced combinations of specific matrices and nanomaterials can mediate and finely tune such stimuli and cues. In this review, we focus on graphene-based nanomaterials as, in addition to modulating nanotopography, elastic modulus and viscoelastic features of the scaffold, they can also regulate its conductivity. This feature is crucial to the determination and differentiation of some cell lineages and is of special interest to neural regenerative medicine. Hereafter we depict relevant properties of such nanofillers, illustrate how problems related to their eventual cytotoxicity are solved via enhanced synthesis, purification and derivatization protocols, and finally provide examples of successful applications in regenerative medicine on a number of tissues.

## 1. Introduction

**Figure 1:**
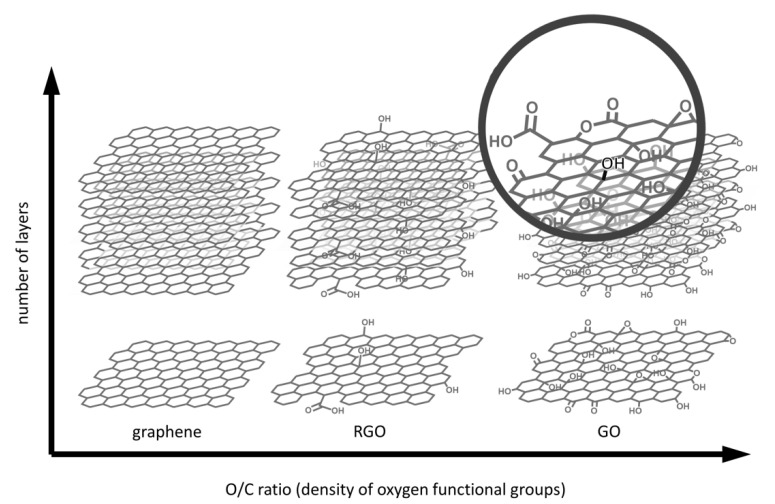
Structural overview of graphene-based materials.

Graphene consists of an atomic honeycomb lattice composed of carbon atoms that can be considered as an indefinite large polycyclic aromatic hydrocarbon with an infinite number of condensed benzene rings. Graphene family is constituted by several derivatives such as graphene oxide (GO), reduced graphene oxide (RGO), graphene quantum dots (GQDs), graphene nanosheets, monolayer graphene, and few-layer graphene [1]. A schematic representation of graphene-based materials (GBMs) taken into account in this review is shown in Figure 1. Although an accurate description of the state of the art in GBM synthesis is out of the scope of this review, a brief outline is provided in Section 2.1. It is vital to stress out that GBMs are highly heterogenous, especially when considering biological properties and applications. Therefore, careful choice of the synthetic method is required to obtain a material with the desired properties (i.e., dimensions, conductivity and eventual functional groups).

Due its high electrical conductivity, mechanical properties and aspect ratio, graphene has become attractive in many fields. In addition to being a rising star in scientific fields other than biology and medicine, graphene, GBMs and composites are widely used for important biotechnological and biomedical applications. Almost all graphene derivatives and composites are being used and tuned to develop special delivery carriers for theranostics [2], gene therapy and drug delivery, and a huge number of examples have been reviewed in recent years [3,4,5,6]. Therefore, we can just list here a few examples of applications in biosensing and bioimaging, before moving to the focus of this review, which is regenerative medicine. 

Conductivity and high transporter capability of graphene allow for tuning biosensor surface features and outperforming many other biosensor types in terms of speed, accuracy, specificity, selectivity and sensitivity. In general, proteins (either catalysts or receptors/ligands) are associated to the graphene-based biosensor surface via electrostatic interaction, covalent bond or by polymer mediated capture. To avoid electrostatic interaction may alter the protein conformation, GBMs are combined with polymers in nanocomposites where mild electrostatics combines efficient binding and maintenance of the original conformation [7]. When instead the conformational dynamics of proteins has to be studied, multilayer graphene nanopore sensors can be used [8]. Several compounds can be detected electrochemically using graphene and GBMs as electrochemical sensor, e.g., cancer markers and cells, ATP, DNA, glucose, toxins, or even proteins [6]. Graphene-based field-effect transistor (FET) biosensors, which can be integrated with electronic chips, easing compatibility with industry standards, are especially applicable in detection of charged molecules such as DNA. Graphene-based fluorescence resonance energy transfer (FRET) biosensors are also widely used with small molecules, nucleic acids and proteins, as reviewed by Zhao et al. [9]. Some biosensors integrate graphene in the surface plasmon resonance (SPR) technology, showing improved sensitivity and detection range [10]. Graphene quantum dots (GQDs) are of special interest to bioimaging in vitro and in vivo because of their biocompatibility, tunable fluorescence with excellent photostability, ultra-small size and hydrophilicity [11]. Stable photoluminescence makes GQDs suitable for cancer bioimaging and has led to biofunctionalization for specific cancer cell imaging and real-time imaging in living cells [12]. GO and RGO are used in bioimaging as well, as their combination with different polymers (e.g. PGA), metal ions or bioimolecules can modulate emissions in three main fluorescence regions (blue, green and red), making (R)GO-derived platforms suitable for multiple tracing and bio-imaging purposes [13,14].

GBMs, and especially GO and RGO find plenty of applications in tissue engineering, where they are employed as scaffolds for tissue regeneration. Tissue engineering is an interdisciplinary technology that gains insights from material chemistry, engineering, cell biology, and immunology to develop biomaterials capable of restoring, maintaining, or improving tissue function or a whole organ [15]. Scaffolds act as biological substitutes that enhance cellular interactions and are able to stimulate the differentiation of stem cells or precursor cells into the desired lineage. The extracellular environment provides biochemical, biophysical, and electrical signals, which all together define tissue-specific niches for proper tissue function and homeostasis. By recapitulating such features in biomimetic scaffolds, the goal of tissue engineering is to guide stem cell development and differentiation to resemble cell organization and behavior in the natural, tissue-specific environment. Such approach offers an interesting translational perspective for tissue repair and regeneration [16,17]. However, successfully reproducing a tissue is extremely challenging since a number of different aspects must be taken into account. In this scenario, nanocomposite materials have proven to be effective in mimicking the required characteristics.

Graphene-based scaffolds (GBSs) are a particular class of scaffold made from graphene, GO and/or RGO nanocomposites. Among the plethora of nanomaterials available, graphene and its derivatives are attractive candidates for developing tissue engineering scaffolds thanks to their tuneable electrical conductivity, excellent mechanical properties, biocompatibility, chemically modifiable surface, and nanoscale dimension matching cell surface receptors and extracellular matrix (ECM) nanoroughness/nanotopography. Morover, they display good capacity to adsorb proteins from the serum (e.g., fibronectin, laminin and albumin), favoring cell adhesion, proliferation and differentiation. 

Graphene structural features and dimensions resemble many components of the extracellular environment such as proteins of the ECM (e.g. collagen), ion channels, signalling proteins and cytoskeletal elements [18]. Therefore, the introduction of graphene or its derivatives into polymeric scaffolds endows them with features that can be tailored to match the ones of the natural tissue of interest. For instance, each tissue has specific mechanical and electrical properties that should be matched by artificial scaffolds. Intuitively, scaffolds for bone regenerative medicine should be stiffer (E > 10^9^ Pa), whereas nervous tissue requires much softer supports (E < 4·10^2^ Pa) and muscles need substrate with intermediate stiffness (E > 10^4^ Pa) [19]. 

Being one of the toughest and strongest nanomaterials discovered so far, graphene incorporation into polymeric scaffolds enhances their mechanical properties, toughness and tensile strength [20]. Therefore, graphene percentage within the scaffold can be modulated in order to better mimic the ECM mechanical properties of the tissue of interest. Moreover, graphene nanocomposite scaffolds are endowed with nanoroughness, which contributes to cell anchoring while modulating cell morphology [18]. This property is particularly important for the differentiation of neuronal cells as graphene establishes tight contact with the growth cone and guides the spreading of developing neurites [21]. Lastly, empirical evidence suggests that engineering the electrical conductivity of the scaffold plays a crucial role in producing a functional electroactive tissue. Since graphene is electrically conductive and its conductivity is stable in biological environments, its incorporation in polymeric scaffolds can reduce the polymer electrical resistance. As a result, graphene-based scaffolds can be used to mimic and regenerate the electroactive tissues like the cardiac and neural ones, but also to boost the repair of non-excitable cells that are subjected to electrical field after an injury, like during bone repair and wound healing [22]. However, Burnstine-Townley and co-workers pointed out that the actual role of scaffold conductivity in cell differentiation is not completely clear. Specifically, disentangling the effect of a single scaffold feature on cell fate can be challenging, as varying graphene content has effect on several properties, such as surface roughness, cellular adhesion and interaction with nutrients, growth factors and wastes [23]. 

At a glance, graphene ability to mimic the natural extracellular environment nanotopography, to retain signalling molecules, to be easily incorporated in both natural and synthetic polymers, and to modulate stiffness and conductivity of the scaffold make it the ideal nanomaterial to provide cues needed to guide cell behaviour and hence an invaluable tool for regenerative medicine applications. Nevertheless, the toxicology profile of graphene and its derivative has not been completely elucidated yet. 

Several drawbacks of GBMs employment for regenerative medicine approaches have been reported which might include membrane damage, hydrophobic interaction, oxidative stress, genotoxicity, mitochondrial disorders and autophagy. However, safety risks should be evaluated case by case based on the intrinsic properties of GBMs, such as purity, surface functional groups, lateral size, stiffness, hydrophobicity and structural defects. Moreover, several reports showed that graphene cytotoxicity is influenced by multiple parameters such as cell population tested as well as graphene dispersibility and functionalization [24,25]. 

## 2. Graphene-Based Scaffolds

### 2.1. Methods for GBM Synthesis

As thoroughly reviewed by Wu and co-workers graphene synthesis can be performed through a plethora of bottom-up or top-down approaches [26]. Among the most common, Chemical Vapor Deposition (CVD), Physical Vapor Deposition (PVD), spin coating, laser ablation and arch discharge needs to be mentioned. Although a systematic review of graphene synthetic methods is out of the scope of this review, it needs to be stressed that the final properties, complexity and cost of a nanomaterial are strictly related to its procedure of synthesis. Each protocol has its advantages and drawbacks, thus the choice should be done taking into consideration the final application of the product. For the sake of clarity, a brief overview of standard approaches is provided in this section.

CVD is often exploited to produce graphene for 2D composites and graphenic foams, described in the next sections. In CVD, gaseous precursors (typically hydrocarbons) are flowed at high temperatures over a metal surface, which acts as a catalyst for their decomposition and leads to the condensation of carbon atoms, forming a graphene sheet. In a typical process, graphene is grown onto a metal surface, supported with a polymer (e.g., poly(methyl methacrylate)—PMMA), and the catalyst is etched by acidic treatment. Subsequently, the graphene foil is transferred on a substrate and the supporting polymer is appropriately dissolved. The choice of metal or alloy for deposition changes process thermodynamics and kinetics, and allows to finely tune the number of graphene layers of the resulting material. The most common metal catalysts for CVD are nickel [27,28] and copper [29], with a preference for the latter due to its capability to produce single- and bi-layered graphene. 

However, bulk production of graphene is more conveniently achieved starting from graphite and weakening the van der Waals forces between its stacked monoatomic carbon layers. Examples for such top-down approaches are liquid-phase exfoliation, surfactant-assisted liquid-phase exfoliation and chemical functionalization. In the first two methods [30,31,32,33,34], exfoliation is achieved through different combinations of factors such as (i) the choice of a solvent with proper surface tension (e.g, γ = 40 mJ m^-2^) [35]; (ii) the use of surfactants, to minimize the interfacial tension between solvent and graphene; (iii) sonication or other external mechanical driving forces; (iv) centrifugation stages to remove thicker graphitic flakes. The principal shortcomings of these methods are the generation of defects and the reduced size attributed to sonication-induced cavitation [36,37].

Among the most common chemical top-down methods, there is the oxidation and subsequent exfoliation of graphite to GO, followed by either chemical reduction or thermal cleavage of oxidized groups to obtain RGO. In a typical procedure, graphite is mixed with sulfuric acid and oxidizing agents in an iterative and synergic action of intercalation and oxidation [38]. Subsequent exfoliation in water then easily yield GO, which can be further modified due its large amount of different oxygen functional groups (such as epoxy, hydroxyl, carbonyl and carboxyl groups). Even if the production of GO induces a large number of defects in the graphenic sp^2^ network, the enhanced hydrophilicity that results from the oxidation can be beneficial for its compatibility in different types of matrixes.

Oxidation is usually carried out at 40–50 °C. However, as demonstrated by Eigler and co-workers, working at lower temperatures could reduce damages to the basal plane. They demonstrated the possibility to synthesize a minimally damaged GO with an almost intact σ framework of C atoms [39] and superior thermal properties [40] while maintaining the oxidation temperature below 10 °C, and effectively controlling kinetics of process.

RGO is obtained from GO with different synthetic methods, yielding materials with different properties. Indeed, thermal treatment (often improperly called “thermal reduction”) and chemical reduction of GO to RGO do not have the same effect on graphene structure, hence on the properties of the resulting materials. The disproportionation induced by thermal treatment of highly oxidized GO brings defective holes in the plane.

Chemical reduction, on the other hand, can be achieved with different reactants [41] leading to different results: as an example, hydrazine leads to *N*-doping in plane, while reduction with L-ascorbic acid leaves adsorbates on RGO that are not easily removed by washing procedures [42]. Since complete reduction of GO is not achieved, RGO differs from graphene due to the presence of residual functional groups; however, O/C ratio of RGO is much lower than that of GO. Even if the sp^2^ network is partially restored, the performances are still lower than those of CVD graphene.

Stability and reactivity of GO are also affected by other parameters such as pH of the dispersion, which is often neglected or underestimated. Indeed, Hirsch and co-workers [43] found evidence that the carbon lattice is damaged by treatments with a base at 40 °C while at 10 °C the partial cleavage of epoxy groups is observed. According to the above-mentioned observations, assessing the O/C ratio, which is often the only parameter considered to assess the successful synthesis of RGO, is clearly not enough to describe the obtained material. It must also be emphasized that the choice of starting materials, different methods of synthesis, and purification procedures have a direct impact on the presence of impurities, that can have a biological effect and can lead to controversial results when materials are used for bio-applications.

**Figure 2:**
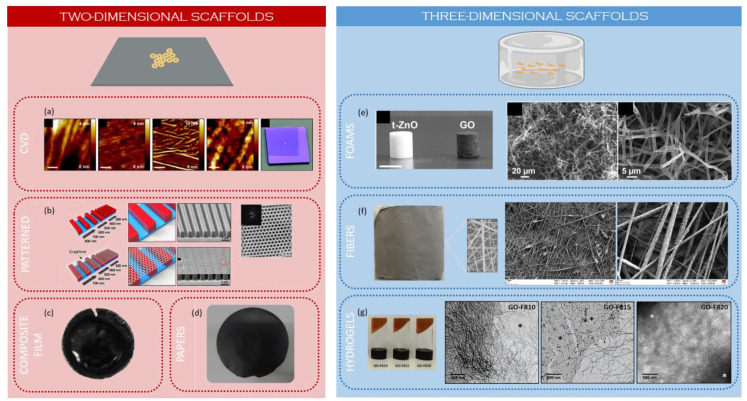
Examples of two-dimensional and three-dimensional scaffolds. (**a**) CVD graphene on Si/SiO_2_ chip and AFM images of graphene transferred on different polymeric substrates [45]; (**b**) graphene transferred on nanopatterned substrate and AFM image [46]; (**c**) PLLA-RGO film obtained as reported in [47]; (**d**) graphite oxide paper [48]; (**e**) GO foams and SEM images [49]; (**f**) PLLA-RGO electrospun fibers obtained as reported in [50]; (**g**) peptide–GO hybrid hydrogels and TEM images [51].

In the next sections, graphene-based nanocomposites will be considered. In these materials, GBMs act as fillers while the matrix is typically an organic polymer (natural or synthetic FDA-approved polymer), though bioglasses and ceramics are also used. In the first part, two-dimensional (2D) scaffolds are discussed, whereas the second part is devoted to three-dimensional (3D) scaffolds (Figure 2). In particular, three types of 3D scaffolds are considered: porous foams, fibrous scaffolds, and hydrogels.

### 2.2. Two-Dimensional Scaffolds

Two-dimensional scaffolds are relatively low cost and easy to fabricate, thus they are often used in preliminary studies to investigate the effect of a specific substrate on cell behavior.

The simplest example of a graphenic 2D-scaffold is represented by CVD-grown graphene (one or more layers) on a PMMA-supported metal catalyst, and then transferred onto a substrate after etching of the metal catalyst. Jangho et al. used this technique to transfer the monolayer graphene onto glass to study its effects on the reciprocal interactions between cells and substrate and to test the possible promotion of human mesenchymal stem cell (hMSC) neurogenesis and neurite outgrowth [44]. In a similar way Nayak et al. transferred CVD-grown graphene on different polymeric substrates to verify the effect of nanotopography induced by interactions between graphene and polymers. Differently from the glass control, their 2D scaffold exhibited nanoripples due to a weaker adhesion, and boosted hMSCs differentiation similarly to treatment with bone morphogenic protein BMP2 [45].

Another method to obtain a graphene-coated surface is based on the chemical modification of a substrate to enable specific interactions with graphene-based materials (GBMs). Ryoo et al. used (3-aminopropyl)triethoxylane (APTES) to decorate the surface of glass coverslips with aminic groups. As a result, they obtained a positively charged surface which could effectively interact with negatively charged GO. Similarly, they exploited (3-glycidyloxypropyl)trimethoxylane (GPTMS) to promote glass interaction with aminated carbon nanotubes (CNT). In vitro tests proved carbon nanomaterial-coated glass to be better at promoting the number and dimensionality of focal adhesions, suggesting good biocompatibility [52].

Two-dimensional graphene-based scaffolds can also be obtained by vacuum filtration of material suspensions. For instance, Jasin and co-workers fabricated graphene-based paper as a substrate for cell growth, air drying vacuum filtrated dispersions of three different starting materials: (i) graphite oxide and graphene oxide with (ii) small and (iii) large average lateral dimensions. Although they did not observe any significant difference on cell adhesion, morphology or proliferation, the smaller release of lactate dehydrogenase (LDH) enzyme compared to control samples, suggested that their scaffold can enhance cell viability [48].

**Figure 3:**
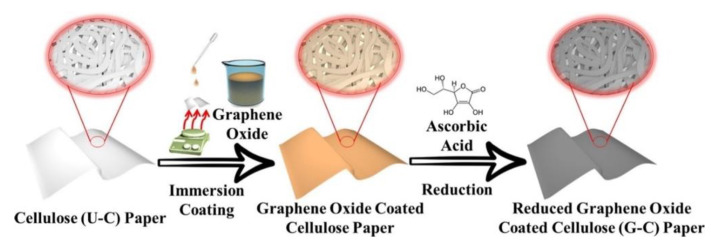
Assembly of RGO-cellulose hybrid paper through deposition of GO followed by in situ reduction [55].

A higher degree of versatility is achieved with hybrid or composite scaffolds, where graphene is used as a filler or coating for polymeric matrices. As an example, Pandele et al. prepared chitosan/GO composites by solution blending, obtaining films with a rough surface useful for cell adhesion. The homogeneous dispersion of GO in a polymeric matrix led to an enhancement of the mechanical properties due to the large aspect ratio of the nanomaterial and its interaction with the polymer chains [53]. Furthermore, Jin et al. tested the viability of a free-standing film composed of GO and bacterial cellulose (BC) obtained from *Gluconacetobacter intermedius*. GO was added to the growth media and *G. intermedius* bio-reduction capabilities were exploited to obtain BC-RGO composites. hMSCs seeded onto these materials showed higher proliferation compared to ones seeded onto films of RGO without the fibrous structure of cellulose [54]. Li et al. fabricated RGO-cellulose paper by drop-casting GO dispersions on cellulose paper, subsequently reducing it with L-ascorbic acid (Figure 3).

These scaffolds showed low resistivity (∼300 Ω/sq), increased mechanical strength and a specific surface micro-topography induced by RGO, which led to improved stem cell adhesion and osteogenic induction. Furthermore, their 2D-scaffolds could be employed with pseudo-3D stacked multilayered constructs that can be configured by rolling or folding, allowing designing a large number of different setups [55].

To enhance their biological effects, two-dimensional scaffolds can be micro- or nanopatterned with specific topographical cues that can direct cell growth and differentiation. Different methods have been developed to this aim, and a pattern can be drawn with either the help of a positive photoresists spin-coated on graphene oxide surface [56], or by transferring CVD graphene on a polymeric nanopatterned substrate [46,57]. This latter approach was adopted by Jangho and co-workers. They transferred a graphene layer on a poly(urethane acrylate)-patterned surface featuring regular parallel nanogrooves, thus obtaining a chemically homogeneous but mechanically heterogeneous substrate. In fact, graphene has lower mechanical properties in regions where it is suspended between nanoridges. Indeed, alignment of hMSCs along the nanotopographical cues of the substrate was observed [46].

Among the plethora of chemical studies presenting new kinds of scaffolds, there is a modest number of works specifically focused on specific GBM functionalization strategies to improve biocompatibility or differentiation capabilities. As an example, Qi et al. functionalized GO with L-theanine, an amino acid that promotes neuronal differentiation. Its presence in a poly(lactic-co-glycolic acid (PLGA) film increased its hydrophilicity and enhanced neuronal differentiation of neuronal stem cells (NSCs) [58]. In our lab we [47,59] designed composite poly-L-lactic acid (PLLA) scaffolds with different carbon nanostructures (CNS) as filler—namely RGO, carbon nanohorns (CNH) and CNT—covalently functionalized with p-methoxyphenyl (PhOMe) groups in order to improve biocompatibility, and the electrical and mechanical properties of materials. RGO- and CNH-based scaffolds (RGO-PhOMe and CNH-PhOMe respectively) showed promising activity in enhancing the expression of myogenic markers during human circulating multipotent stem cell (hCMCs) differentiation. Moreover, electric percolation was found to take place within the considered range of RGO concentration, tough with lower performances compared to CNT-based samples. This difference is likely due to the influence of aspect ratios on electrical behavior.

Despite the aforementioned potentialities, 2D scaffolds have limitations. First of all, a two-dimensional environment is not suited to reproduce natural ECM. Then, nutrients are directly available to cells and wastes can diffuse to a limited extent. Lastly, altered cell–cell interactions may result in unpredictable cell responses. Therefore, in recent years the focus has shifted towards the study and design of 3D-scaffolds in order to overcome these limitations.

### 2.3. Three-Dimensional Scaffolds

As already mentioned, 3D scaffolds recapitulate tissue biophysical features thus are better candidates for in vivo applications. Scaffolds with a three-dimensional architecture should be endowed with a highly interconnected porous network. Recently, Lutzweiler and co-workers reviewed the effects of porosity, pore size and shape, interconnectivity and curvature in scaffolds used for tissue regeneration: not only these properties directly influence migration of nutrients and wastes inside the scaffold, but also the permeation and communication between cells [60]. Recent evidence suggests that scaffolds with pore diameters between 100 and 750 µm are generally beneficial while larger pores make cells experience a planar pseudo-2D environment, which differs from their natural environment [61,62].

#### 2.3.1. Foams

The easiest method to fabricate porous scaffolds involve freeze-drying filtrates or suspensions. For example, Domínguez-Bajo et al. produced RGO foams by drying GO slurries, obtaining structures with 43% of porosity and 30 µm of pore size after thermal reduction. In addition, these scaffolds had a relatively low Young’s modulus (~1.3 kPa) and made a good candidate for nervous tissue engineering. When their applicability on neural repair after spinal cord injury was tested in vivo, not only scaffolds were populated by nerve cells, but the authors also observed full vascularization [63]. In another instance, the same group exploited ice segregation-induced self-assembly, based on unidirectional freezing of dipped suspensions and lyophilization, to fabricate hierarchically channeled RGO scaffolds with controlled porosity and pore size (80% and 150 µm respectively) [64]. Liao et al. exploited a freeze-drying approach to produce a porous hybrid scaffold based on a copolymer composite of methacrylated chondroitin sulfate (CSMA) and poly(ethylene glycol) methyl ether-ε-caprolactone-acryloyl chloride (PECA) with GO, synthesized by heat initiated free radical polymerization. Not only scaffolds pore size could be tuned by CSMA:PECA ratio, but the compressive strength increased with PECA content, with values consistent with cartilage tissue. The plateau limit of conductivity (1.84 S/m) resulted at 3% GO content [65].

In a similar way, Hermenean et al. fabricated a porous chitosan/GO scaffold with improved mechanical performance—i.e., increased compressive strength and tunable Young’s modulus while keeping scaffold flexibility—observing that the incorporation of 3% of GO significantly enhanced bone regeneration in vivo, compared to pure chitosan scaffolds, even in the absence of additional differentiating agents, confirming the active action of GO in facilitating cell infiltration and differentiation [66].

Graphene foams are the first porous structures composed of single layer graphene, applied in tissue engineering. Besides porosity, these scaffolds are endowed with a wrinkled topography induced by the synthetic process, which is beneficial for cell adhesion and proliferation since it better mimics the ECM [67,68]. Li et al. compared NSCs differentiation performance on 2D CVD graphene scaffolds and 3D graphene foam and observed improved proliferation and differentiation towards mature phenotypes on the latter substrate [69].

**Figure 4:**
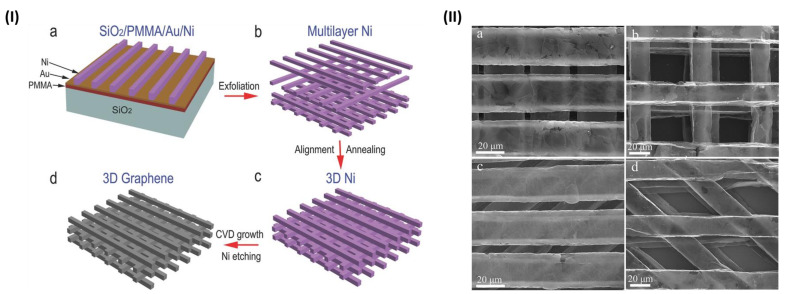
(**I**) (**a**–**d**) Schematic illustration of the procedure used to fabricate a 3D-CG. (**II**) (**a**–**d**) SEM images of four layer freestanding 3D-CGs with different patterns, pores, and skeleton sizes [70].

In the techniques described so far, pore size and interconnectivity depended on the Ni foam features. However, Xiao et al. recently managed to finely tailor these properties, fabricating an ordered architecture of Ni: they used photolithography to define a mask in which Ni was deposited by electroplating and aligned. Graphene was then grown through CVD on the resulting Ni template (Figure 4). Thanks to this procedure, they managed to design a scaffold with defined features by tuning pore and skeleton size (10–50 μm range), orientation angles (45° or 90°), electrical conductivity (60–80 S cm^−1^ range) and density (around 3–4 mg cm^−3^). Such a scaffold was able to direct neuronal growth and align neurons along a defined path to form a network [70].

Another method to fabricate porous structures has been employed by Rasch et al. [49]. Starting from tetrapod-shaped ZnO, pressed and annealed in a mold, they were able to synthesize templates with high porosity (50 to 98%). GBM deposition was obtained by infiltrating a GO suspension in the templates, followed by chemical etching with hydrochloric acid. Their protocol allowed easy, versatile and cost-effective deposition of nanomaterials. Moreover, biological evaluations of these scaffolds by Schmitt et al. showed they could be promising for nervous tissue engineering [71]. 

An alternative approach to induce porosity in scaffolds is supercritical foaming which allows to control scaffold morphology through a careful choice of experimental parameters, such as chamber pressure, temperature and decompression rate. Evlashin et al. exploited this process to manufacture RGO-reinforced polycaprolactone (PCL/RGO) and PCL/GO foams in a carbon dioxide atmosphere. Although the presence of RGO in the polymer matrix led to an increase of pore size, those foams showed poor cell adhesion properties. Conversely, they found PCL/GO scaffolds to enhance cell adhesion. However, both composites displayed lack of interconnected porosity, resulting in cells attaching only on scaffold surface [72]. Polymer-enriched hybrids can also be obtained starting from CVD graphene foams, by depositing the polymer from a solution through spin or dip coating. Resulting scaffolds show improved mechanical performances and cellular responses. In order to retain porosity, it is crucial to avoid pore saturation through fine optimization of dip coating time and by choosing a polymer with a favorable, near zero contact angle. Nieto et al. exploited this technique with a copolymer of polylactic acid (PLA) and poly-ε-caprolactone (PCL) and achieved improved tensile strength due to filling of the pre-existing microcracks in pristine G foams. In vitro tests demonstrated these materials are able to support hMSCs viability and differentiation, making them suitable for musculoskeletal tissue engineering [73].

A layer-by-layer (LBL) technique was followed by Song and co-workers who deposited a positively charged polymer, poly(diallyl dimethylammonium) chloride (PDDA) on a negatively charged Ni template and subsequently placed negatively charged GO onto its surface, which was then thermally converted to RGO. Electrochemical deposition of polypyrrole (PPY) and hydroxyapatite (HA) on top, increased scaffold roughness and surface area, favoring cell adhesion and proliferation as confirmed by *in vitro* tests on the pre-osteoblast cell line MC3T3-E1 [74].

Besides metallic templates, polymeric organic foams are used for polymer replication technique, especially in the inorganic scaffold field. Deliormanlı et al. used polyurethane foam to fabricate HA scaffolds, eliminating the template and sintering HA by heat treatment. PCL/GO was added by dip coating, leading to a scaffold with improved mechanical performance and higher bioactivity [75]. 

The same procedure can be applied to bioactive glass, another important class of useful scaffolds in tissue engineering. As an example, Turk et al. incorporated 10% graphene directly in the glass matrix before sintering borate-based porous scaffolds, doubling the compressive strength and obtaining an electrical conductivity (0.060 S/cm) which could be exploited to electrically stimulate cell growth [76]. Moreover, Deliormanlı et al. fabricated more chemically stable and biocompatible silicate-based scaffolds coated with PCL/graphene with pore size between 100–500 μm, without detrimental effects of polymer coating on pore structure [77].

An alternative approach to porous structure design is 3D printing, which allows to accurately control scaffold geometrical features without the limitation of using a template. Jakus et al. exploited a PLGA-based ink where they incorporated graphene with the use of surfactants and plasticizers. The mechanical properties of composites are affected by graphenic particles, with an increase of elastic modulus to a value of 16 MPa at 20 vol% loading of graphene, but with detrimental effects at higher loadings (40–60 vol% of graphene). In addition, they observed an anisotropic alignment of graphene flakes, enhancing electrical conductivity due to shear forces produced during the 3D printing extrusion process, which increased with the decrease of the nozzle diameter [78]. 

Cabral et al. used extrusion 3D printing to produce multicomponent scaffolds, based on tricalcium phosphate chitosan and gelatin, which mimicked the inorganic and organic components of bones, respectively. GO was added to this blend and reduced to RGO *in situ* by L-ascorbic acid treatment. When comparing mechanical properties of scaffolds incorporating GO or RGO they found the latter to better mimic bone Young modulus, thus their scaffold might be useful as a temporary support for bone regeneration [79].

#### 2.3.2. Electrospun Fibers

**Figure 5:**
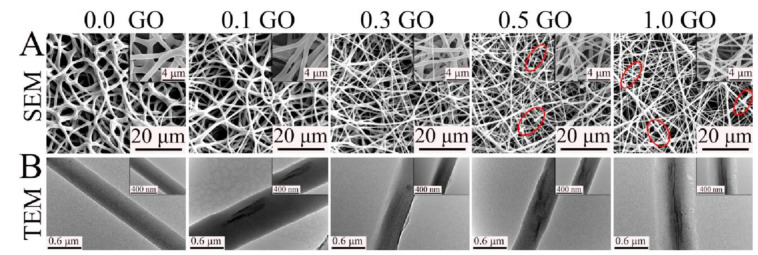
Surface morphological and constructional images of PCL/GO composite nanofibers with different GO concentrations (wt%): (**A**) SEM images and (**B**) TEM images. The red ellipses in SEM images are the fractures [84].

Fiber-based scaffolds are largely employed in tissue engineering because they intrinsically resemble the microstructure of natural tissues. Fiber diameter, porosity, and orientation are the main features that influence cell growth and tissue regeneration [80]. One of the most common techniques to produce continuous fibers is electrospinning. Electrospinning offers several advantages, including (i) ease of processing, (ii) possibility of large-scale production (iii) availability of advanced modes [81]. Moreover, it is highly versatile and electrospun fibers can be deposited in a random orientation or in an aligned fashion which enhances cell alignment and elongation along the contacted fiber direction [82]. Most thermoplastic materials can be electrospun by fine-tuning the properties of the polymeric solution and the electrospinning parameters such as voltage, electrodes distance and flow rate. The American Food and Drug Administration (FDA) approved several thermoplastic biomaterials for in vivo implantation. Nevertheless, their applications are restricted by the high hydrophobicity, low mechanical properties, lack of specific interactions with cells and sometimes relative slow in vivo degradation rate. Luckily, these limits can be easily overcome by introducing proper nanofillers, and several examples of electrospun thermoplastic materials reinforced with GBMs have been reported [83,84,85]. As highlighted by Song et al., the solubility of the filler strongly influences the mechanical properties of the final material: a poor dispersibility or a too-high loading leads to aggregation, which results in fractures and disconnections along the nanofibers. It has been observed that electrospinning graphene-based composites yields thinner fibers (Figure 5), but on the other hand, even a small amount of GO or RGO inside the electrospun fibers reinforces their structure and overcomes the detrimental effect of a reduced diameter on mechanical properties. Therefore, it is crucial to finely tune the CNS content in order to find the right balance between a uniform CNS dispersion, nanofiber diameter and reinforcement effect. Besides, it has been widely demonstrated that incorporation of CNS in fibrous scaffolds results in an improved biomimetic microenvironment that enhances cell adhesion and proliferation on different cell types [84].

Generally, the smaller thickness induced by graphene-based nanofillers on electrospun fibers allows mimicking the structure of ECM even better. It is believed that the effect of GBMs on fiber diameter is due to the electrical conductivity of the feeder solution, which is a key factor in determining the diameter and size distribution of the electrospun fibers. Moreover, it is reported that fiber diameter is highly correlated to the viscosity of the feeder solution. Scaffaro and colleagues pointed out that the decrease of viscosity of a PCL solution by addition of GO induces electrospinning of thinner fibers. On the other hand, they observed an opposite effect on viscosity (and fiber diameter) with GO-grafted-PEG (GO-g-PEG). Functionalization of the filler not only increased fiber diameter, but also improved dispersion of the filler and maximized the filler/matrix interfaced area, making GO-g-PEG more effective than GO in reinforcing composite fibers, in particular at low concentration [86].

In 2019, Basar and co-workers developed a PCL/GO composite scaffold [87] by functionalizing GO with either an RGD-peptide (GRGDSP), thiophene (Th) or both. Besides having the aforementioned effect on fiber diameter, GO functionalization yielded an enhanced electrical behavior to the scaffold, with conductivities reaching 15.06 µS cm^-1^ in PCL/GO-GRGDSP-Th (2% of GO), a 15-fold increase compared to neat PCL (0.95 μS cm^−1^). However, while scaffolds with higher content of GO (2%) showed higher electrical performances, the elastic modulus and tensile strength of 0.5% GO-scaffolds were found to be higher. Once again, this result was associated with the uniform dispersion of GO in the polymer matrix. Interestingly, this modification resulted in an increment of both electric conductivity and mechanical stability due to the ability of sulfur moieties to enable the crosslink between GO and PCL [87]. 

Scaffold properties can also be altered by combining different organic or inorganic fillers. Lui et al. developed electrospun PLA scaffolds reinforced with GO (1–3 wt %) and/or nano-HA (15 wt %). Interestingly, addition of 15 wt % nano-HA improved both elastic modulus and tensile strength, whereas concentrations of GO above 2% diminished them due to filler aggregation. Nanofiller addition slightly increased scaffold glass transition temperature and modified the hydrophobicity of PLA, enhancing the polymer water uptake, which in turn assisted cell adhesion and proliferation [88].

Different strategies have been developed to obtain polymeric nanofiber scaffolds based on graphene and its derivatives. However, nanocomposites fail to provide a pure graphene interface. An alternative approach aims to immobilize nanostructures on the surface of polymeric nanofibers. The surface of aliphatic polyesters such as PCL and PLA can easily be functionalized with hydroxyl and amino groups by treating the polymeric scaffold with a diamine solution. In tissue engineering, aminolysis of polyesters improves their interactions with cells and allows them to form a stable graphenic coating [89]. Recently, Jalili-Firoozinezhad et al. reported an easy method to generate electrically conductive nanofibers by coating a PCL nanofibrous mat with GO liquid crystals, which were then reduced to RGO to form PCL-templated graphene nanofibers [90]. Proper electrical conductivity and nanofibrous topography of these constructs make them an ideal platform for cell culture, tissue engineering, drug delivery, and biosensor applications. Preliminary in vitro analyses using hMSCs revealed no induced cytotoxicity and confirmed an enhanced cellular metabolism and proliferation rate compared to standard culture plates and PCL nanofibers.

Indeed, coated fibers can be obtained without any surface treatment. Wang et al. developed a conductive graphene-based fibrous scaffold by coating RGO via an in situ redox reaction of GO on the surface of silk fibroin/poly(L-lactic acid-co-caprolactone) (ApF/PLCL) composite nanofibers [91]. The authors highlighted that the coating did not affect the nanoscale topography of the scaffold and enhanced its mechanical properties, electroactivity and biocompatibility. They then investigated how these conductive scaffolds regulated in vitro and in vivo cell behavior and differentiation under electrical stimulation. RGO-coated ApF/PLCL scaffolds boosted cell migration, proliferation and myelin gene expression of Schwann cells (SCs), whereas pheochromocytoma-derived PC12 cells cultured on these scaffolds exhibited enhanced differentiation. In vivo implantation of the constructs promoted peripheral nerve regeneration in rats.

Polymer core-CNS shell fibers can be obtained by electrospinning the polymer into a solution of graphene or one of its derivatives. Subsequently, it is possible to further functionalize or reduce the shell. Jin et al. exploited this principle to develop an RGO core-shell nanofiber (RGO-CSNFM) [92]. The RGO core-shell structure displayed high mechanical, electrical conductivity (10.0 S cm^−1^) and a charge carrying capacity. This property is likely due to both RGO-CSNFM large surface areas and the extended π–π conjugated bond network generated over the surface of the RGO shell layer. Wu et al. developed an LBL method to coat electrospun nanofibers that mimic vascular ECM and enhance proliferation of endothelial cells. PLLA surface modification was achieved via electrostatic LBL self-assembly by alternately immersing PLLA fibers in a positively charged solution of 0.1 wt% chitosan and a negatively charged solution of 0.1 wt% heparin (PLLA-CS/Hep) or 0.1 wt% heparin/graphite oxide (PLLA-CS/Hep/GO). After the LBL coating, the hydrophilicity and mechanical properties of the modified PLLA nanofibers were greatly enhanced. Moreover, the CS/Hep/GO coating positively influenced cell attachment, viability, and proliferation of endothelial cells [93].

The versatility of electrospinning allows to obtain complex and ordered structures. A compelling example has been reported by Shao and co-workers [94] who used electrospinning to develop a 3D scaffold with multiple orthogonal aligned fibers. This peculiar architecture improved mechanical properties and decreased issues that may arise when working with parallel fibers or random networks. Moreover, a 3D structure better mimics the natural cellular environment. They developed an electrospun PLGA/silk fibroin/GO/hydroxyapatite (PLGA/TSF/GO/HA) 3D scaffold. hMSCs seeded onto these scaffolds showed enhanced proliferation and elongated morphology along the long axis of the nanofibers. Lastly, biological assays indicated that composite scaffolds enhanced osteogenesis and alkaline phosphatase activity.

In another work, Zhang and co-workers combined GO nanosheets and aligned aminolyzed PLLA nanofibers which favored nerve regeneration. The aminolysis of PLLA nanofibers allowed to form a stable GO coating. Schwann cells (SCs) cultured on these nanocomposite scaffolds displayed improved proliferation and elongation along the fiber direction compared to those grown on the aligned PLLA and aminolyzed-PLLA. The coated structure was also able to improve differentiation and neurite outgrowth of pheochromocytoma derived PC12 cell line. The authors suggest that these results may arise from the modification of surface chemistry and roughness induced by the GO coating [95].

#### 2.3.3. Hydrogels

Hydrogels are three-dimensional entangled networks able to retain large amounts of water. Despite being mostly liquid, they display a solid-like rheological behavior and recently they have been employed as scaffolds for tissue engineering [96,97]. Hydrogels can be categorized into two main classes based on the forces involved in building the network: (i) chemical and (ii) physical gels. The network of chemical hydrogels is obtained through covalent cross-linking of its components, which generates a permanent structure. On the other hand, the structure of physical hydrogel is characterized by reversible non-covalent interactions which make these gels suitable for cell encapsulation but highly susceptible to environmental conditions (i.e., ionic strength, pH, temperature), such that even minor changes can cause the network to collapse. Indeed, physical hydrogels exhibit lower mechanical properties than their chemical counterpart. However, even chemical gels generally cannot withstand high mechanical stress despite the covalent cross-links [98]. 

Graphene and graphene derivatives in hydrogels may play the role of (i) self-assembling gelator molecule or (ii) filler in order to prepare multi-functional nanocomposite hydrogels. Self-assembly has been recognized as one of the most effective “bottom-up” strategies for building structured networks. Driven by non-covalent π-π interactions that arise from their 2D structure, graphene and graphene derivatives spontaneously re-organize into a 3D structure. Self-assembled hydrogels can be prepared through a one-step hydrothermal method starting from a graphene-based solution [99]. For example, Yang and colleagues have demonstrated the jellification of GO at the solution–filter membrane interface, creating highly conductive and anisotropic films [100]. 

The employment of pure graphene and/or graphene derivatives hydrogels is quite restricted, thus they are mainly used as high-quality nanofillers for composite hydrogels [101]. Different synthetic and natural polymers able to form hydrogels are suitable for tissue engineering scaffolds. Among synthetic polymers we may mention polyethylene glycol (PEG), poly(acrylamide), poly(lactic acid) or synthetic peptides. Natural-derived polymers such as alginate, chitosan, collagen, silk or gelatin are also widely used to fabricate hydrogel scaffolds for tissue engineering. Polymeric scaffolds display good biocompability and biodegradability but lack, for example, the ability tolerate strong mechanical forces [102,103]. 

Alginate is a natural polysaccharide composed of β-D-mannuroic acid (M) and α-L-guluronic acid (G) typically obtained from brown seaweed. In the presence of various divalent cation (Ca^2+^, Mg^2+^), alginate polymers form gels via non-covalent cross-linking of the carboxylate groups of the G blocks on the polymer backbone. Even if the concentration of crosslinker, percentage of G content and jellification time allows to tune the properties of alginate hydrogels, other limitations cannot be overcome without the use of specific fillers. Particularly, alginate-based hydrogels do not permit good control over their internal architecture, they lack cell receptors adhesion sites and suffer from low protein adsorption capability [104]. As independently highlighted by Losic et al. and Chen et al. [105,106], the introduction of GO and RGO in an alginate matrix allows to modify and control the porosity of the gel (ca. 99%±0.3%), making the pores size uniform from surface to its inner core and fostering cellular activity. GO and RGO composite gels also allow to reach the optimal swelling index required for an efficient scaffold. Investigation of mechanical and electrical properties revealed an optimum GO content of 0.1 wt%. Above this concentration a detrimental effect was observed due to an imperfect dispersion of GO within the alginate matrix.

Chitosan, as well its derivatives, is a widely available natural polymer characterized by excellent biological properties (i.e., biocompatibility, coagulation activity, biodegradability). Agarose (AG), on the other hand, is a polysaccharide obtained from red algae, displays a thermo-sensitive behavior and exhibits mechanical properties similar to that of soft tissues. However, its employment is limited by the lack of cell recognition sites. 

**Figure 6:**
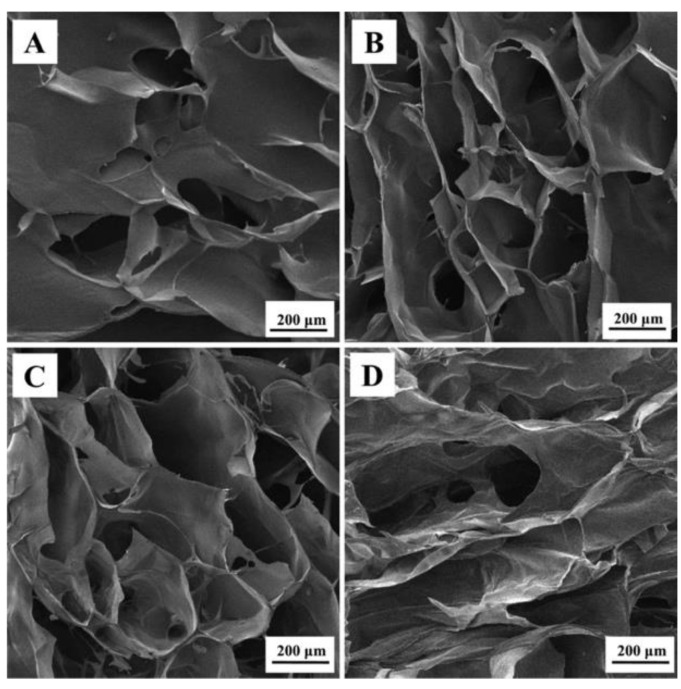
SEM images of (**A**) AG/CS (0% GO), (**B**) AG/CS/GO (0.5% GO), (**C**) AG/CS/GO (1% GO), and (**D**) AG/CS/GO (1.5% GO) composite scaffolds [107].

Sivashankari and Prabaharan used GO as a nanofiller for the fabrication of agarose/chitosan (AG/CS)-based scaffold [107]. Through a freeze-drying method, they prepared 3D AG/CS/GO scaffolds with different concentrations of GO (0–1.5 wt %). GO introduced changes in the scaffold morphology, in their swelling behavior and in their water retention ability. In particular, AG/CS/GO scaffolds with 1 and 1.5 wt % of GO exhibited the highest porosity (Figure 6), with an average pore size (237–274 μm) matching the demands for bone tissue regeneration [108,109]. Even with the increase in porosity, GO likewise enhanced the mechanical properties due to interactions established between fillers and polymer matrix and favored cell attachment and proliferation. Freeze-drying techniques are widely employed for scaffold generation and also allows to obtain anisotropic scaffolds. Liu et al. developed a highly oriented hydrogel through directional freezing of CS/GO suspension on a copper plate cooled with liquid nitrogen [110]. This method produced micro-sized ice rods within the suspension, which act as template for a honeycomb-like structure resembling a bone lamellae structure. The resulting hydrogel displayed anisotropic mechanical behavior improved by the incorporation of GO and were able to guide the growth of mouse osteoblastic MC3T3-E1 cells along the longitudinal direction of the honeycomb structure.

Self-assembling peptide-based hydrogels (SAPHs) have been widely employed as vehicle for drug delivery, but they can also be employed in tissue engineering due to their biocompatibility and non-immunogenic nature [104,111,112]. Ligorio et al. used GO as nanofiller in a peptide (FEFKFEFK) hydrogel for tissue engineering [51]. After conditioning with cell culture media (i.e., pH 7.4), all gels displayed an enhanced storage modulus. Bovine nucleus polposus (NP) cells were cultured on these hydrogels to assess cell viability and GO-hydrogels with shear modulus similar to the native NP showed higher viability and constant metabolic activity throughout the culture period. 

Wang et al. prepared a silk fibroin scaffold incorporating exfoliated graphene [113]. An aligned silk fibroin hybrid hydrogel was obtained by application of an electric field. Even if aligned silk nanofiber gels were previously proven to be able to influence behavior such as cell orientation and migration [114], they failed to actively induce neural differentiation. The nanocomposite hydrogels displayed anisotropic mechanical properties, and the one with the highest content of graphene showed doubled parallel and orthogonal compressive moduli compared to graphene-free samples, making them suitable for nerve tissue engineering. After the addition of graphene, cell proliferation was further enhanced, indicating that graphene sheets effectively induced neurite differentiation.

In recent years, injectable hydrogels have drawn major attention since they need minimal invasive procedure to be administered and have reduced therapeutic costs. The hydrogel precursor should be injected as a controllable liquid (i.e., characterized by low viscosity) and must jellify into a robust hydrogel as quickly as possible *in situ* [115]. Finally, it is uttermost important that gelation occurs after injection and at physiological conditions (temperature and pH). Recently, Lee et al. developed an injectable GO-incorporated glycolchitosan-oxidized hyaluronic acid (gCS/oHA) hydrogel [116]. Gelation of gCS/oHA was obtained through the cross-link between the aldehyde group on oHA and the amine groups of gCS (Schiff-base reaction). Frequency sweep experiments were used to investigate the mechanical properties in a plate–plate geometry. The results showed that when the GO content increased, the G’ value gradually increased too, suggesting a more robust hydrogel formation. GO may enhance polymer cross-linking through hydrogen bonding interactions [117,118]. GO-incorporated hydrogels displayed lower cytotoxicity and higher osteogenic activity compared to control both in vitro and in vivo. High levels of COL1 expression observed in cultures hinted that these injectable gels could be suitable for treating bone injuries. Saravanan et al. explored chitosan-glycerophosphate-based injectable hydrogels for treatment of bone defects [119]. Due to newly introduced non-covalent interactions, GO (0.5% *w*/*v*) composite hydrogels significantly increased swelling, protein adsorption and cell interaction compared to their GO-free counterparts. Moreover, GO introduction reduced gelation times and controlled degradation rates.

Poor dispersion of GBMs within the polymer matrix causes aggregation [105], which may be detrimental for scaffold properties. To achieve homogeneous and stable dispersions of GBMs, covalent and/or non-covalent functionalization may be required. Díez-Pascual et al. [120] fabricated poly(propylene fumarate) (PPF)-based nanocomposites reinforced with GO, non-covalently functionalized with PEG (PEG-GO). PEG functionalization reduces the aggregation tendency and cytotoxicity of GO without impairing its unique features. The presence of PEG-GO leads to a threefold increase of Young’s modulus at 3% loading of filler and improved cell adhesion and growth. The results have been ascribed to the roughness of the scaffold, the hydrogen-bonded network established between GO and the polymer and the good GO dispersion inside the matrix. Polymers may be also covalently bonded to GO, for example by esterification. Noh et al. designed a graphene oxide GO covalently functionalized with acrylated polyethylene glycol (PEGA-GO) through ester formation [121]. The PEGA-GO was photopolymerized with polyethylene glycol diacrylate (PEGDA) leading to gel formation. GO-doped hydrogels boosted cell adhesion and osteogenic differentiation, though no changes were observable in swelling and mechanical properties.

Wu et al. took advantage of the abundant functional groups on the surface of nanosized GO to link starch chains via esterification [122]. Starch is a widely available and cost-effective polysaccharide, which does not release degradation products that induce inflammations in vivo. Nanosized GO was synthesized from starch through microwave-assisted degradation and then covalently bonded to the polysaccharide itself to improve its mechanical features and bioactivity. In another example, Ruan et al. crosslinked carboxymethyl chitosan (CMC) to GO by amide bond formation [123]. The obtained GO-CMC scaffolds appeared rougher than their GO-free counterparts and showed better retention properties and slower degradation rates thanks to the higher cross-linking degree compared to the GO-free and CS/GO-CS samples. Water uptake and retention rates are important parameters, since the scaffold is the vessel for nutrients and metabolites for cell activity. The authors highlighted that GO introduction also deals with the poor mechanical strength typical of bare CMC [124].

## 3. Stem Cell Differentiation and Mechano-Transduction

### 3.1. Tissue Engineering and Stem Cells

**Figure 7:**
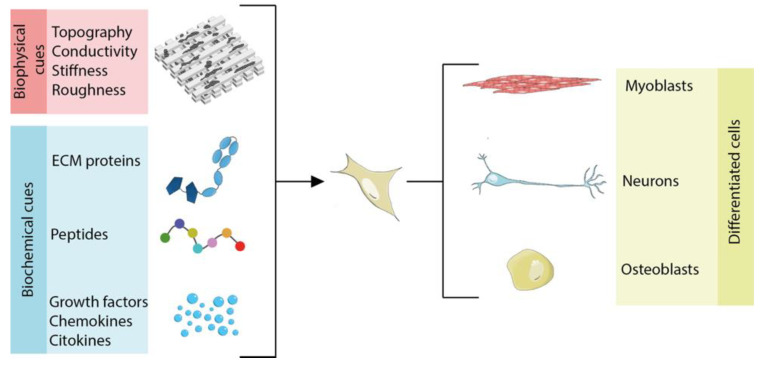
Schematic representation of biophysical and biochemical cues on cell differentiation.

Stem cells are non-specialized cells with self-renewal potential and the ability to differentiate into various cell types if directed with appropriate stimuli, making them a powerful tool for the regeneration of injured tissues [125]. Embryonic Stem Cells (ESC) are pluripotent stem cells able to originate all the cell types of the body [126]. Despite their ideal self-renewing capabilities and differentiation potential, they are not widely used for tissue engineering studies due to the ethical restrictions of human embryo use in research. As a valid alternative, tissue engineering switched the focus to adult stem cells, which are stem cells residing throughout the body whose role is to maintain and repair the tissue in which there are found. Such cells have a limited differentiation potential compared to ESCs but offer the advantage of being isolated directly from the patient for autologous regenerative therapies. Good examples of adult stem cells are the Mesenchymal Stem Cells (MSC) and Hematopoietic Stem Cells (HSC). They both can be isolated from patient bone marrow and can regenerate bone, cartilage, and adipose tissue (MSCs), as well as the entire immune system (HSCs) [127,128]. However, some adult stem cells, like neuronal stem cells (NSCs), can be isolated only with very invasive procedures and in small quantities [129]. As of now, the most promising stem cell type for regenerative applications are the induced Pluripotent Stem Cells (iPSCs). iPSCs are generated from the “reprogramming” of somatic cells back to the pluripotent “embryonic” state [130]. Therefore, they show the same “unlimited” self-renewal and differentiation capabilities of the ESCs, with the advantage of being free from ethical issues as reprogrammed from patient or donor-matched somatic cells [131]. Challenges associated with the iPSC clinical use are (i) the difficulties in finding HLA-matched donors (especially for mixed-race patients) and (ii) the time and costs for the development of patient-derived iPSCs, particularly considering the extensive validation and stringent regulatory processes that would require each patient-derived cell line [132,133]. However, recent works proposed new strategies to engineer such iPSCs to make them “invisible” to the recipient immune system, showing that we are very close to the generation of off-the-shelf, universally compatible iPSCs for the allogenic treatment of a myriad of diseases [134,135,136]. The interaction between stem cells and the extracellular microenvironment is critical in controlling stem cell differentiation, as depicted Figure 7.

### 3.2. Cues Controlling Stem Cell Behavior

Biochemical cues are provided by reciprocal interactions between the cell, soluble bioactive agents, and the ECM. Soluble molecules, such as growth factors, chemokines, and cytokines, diffuse to bind the cell surface receptors and have potent effects on cell growth, proliferation, and differentiation. Insoluble ECM macromolecules (e.g., collagens, elastin, and laminin), glycoproteins (e.g., fibronectin and vitronectin), and polysaccharides (e.g., heparan sulfate and hyaluronic acid) form a meshwork of fibers or fibrils with ECM glycoproteins incorporated into them. The resulting matrix is tissue-specific and functions as both a structural and signaling scaffold to cells [137].

Many works showed that some of the aforementioned molecules—if administrated both in vitro and in vivo—are able to elicit specific cell responses [138]; moreover, different strategies have been developed to link such proteins to biomaterial scaffolds in order to help delivery at the injured sites [139]. However, coating surfaces with recombinant proteins or native matrix macromolecules extracted from animal tissues encounters the problem of eliciting immune responses, in particular when using proteins from different species. Furthermore, their isolation and purification from native tissues or their production as recombinant proteins at a larger scale for tissue engineering purposes is expensive and subject to batch-to-batch variability [140]. For these reasons, the production of specific motifs known to mediate regulatory signals as synthetic peptides presents significant advantages compared to using entire recombinant/native tissue proteins: (i) low immunogenic activity; (ii) increased stability; (iii) low production costs; and (iv) simplified preparation and immobilization onto substrates. Moreover, peptides can be: (v) presented to cells at surface densities significantly higher than those possibly achieved with entire proteins or domains; and (vi) tailored in composition for each tissue-specific application [141]. The biomimetic peptides most used for scaffold functionalization are the ones representing the ECM protein epitopes for integrin binding and therefore promoting cell adhesion [142]. In addition, tissue-specific peptides, resembling active motifs of growth factors and transmembrane proteins, have also been used to tune the cell differentiation [143,144].

Cells are capable of sensing and responding to biophysical cues, over a wide range of length scales. Many of these cues are provided by the ECM, which acts as a cellular scaffold and is the primary extracellular component in tissues. In vivo, the ECM, through its structure and molecular composition, presents a variety of geometrically defined, three-dimensional (3D) physical cues in the submicron to micron scale, referred to as topographies. Cell response to topographies is mediated by a phenomenon called contact guidance, which is known to affect cell adhesion, morphology, migration, and differentiation [145]. Another physical cue displayed by the ECM is mechanical stiffness through which, similar to topography, a diverse set of cellular functions can be modulated. Matrix sensing requires the ability of cells to pull against the matrix and cellular mechano-transducers to generate signals based on the force that the cell must generate to deform the matrix. Mechano-sensitive pathways subsequently convert these biophysical cues into biochemical signals that commit the cells to a specific lineage [145]. For example, MSC differentiation can be modulated by substrate stiffness [146], while developing neurons are able to transduce topographical stimuli through the interaction of the growth cone with the immediate environment. Such mechanical cues direct neurite extension, ensuring appropriate and regulated connectivity within the overall neural circuitry [147].

ECM mimicry can be achieved using either natural or synthetic polymers interconnected by physical and ionic interactions and even covalent linkages [148]. Electrospun polymer fibrous substrates with controlled fiber architectures and diameter provide topographical cues to cells by presenting geometries mimetic of the scale and 3D arrangement of the collagen and laminin fibrils of the ECM. Such polymer fibers present a high surface-to-volume ratio and porosity and are hence well-suited for promoting cell adhesion, growth, and differentiation and enable growth factor/drug loading; such properties are inherent to bioactive matrix microniches [50,149]. Recent advances in 3D bioprinting strongly improved our ability to imitate natural features of ECM. As an additive manufacture technology, the 3D bioprinting allows deposition of polymers, hydrogels, cells, growth factors, and peptide active motifs by using a layer-by-layer approach to build up arrangements favorable to tissue-like structure formation, which are endowed with superior differentiating properties compared to the conventional 2D culture vessels [148].

Endogenous electrical signals are present in many developing systems and influence crucial cellular behaviors—such as cell division, cell migration, and cell differentiation [150]. Some cell types, like osteoblasts, neurons, and cardiomyocytes, are especially sensitive to electrical signals as they activate membrane receptors and downstream intracellular signaling elements leading to specific cell responses [151]. Not only cells, but also extracellular matrix proteins, such as collagen, fibrin, and keratin, can generate electrical currents upon mechanical stress, a phenomenon known as piezoelectricity [152].

Electrically conductive scaffolds not only enhance path finding of growing axons [153], but also improve cell survival and functional integration after transplantation in vivo by providing structural support for transplanted cells and facilitating synaptogenesis with host cells by restoring the neuronal network activity [154,155].

Since electroactive myocytes are responsible for heart and muscle contraction, electrically conductive materials found applications in cardiac and muscle tissue engineering as they support and maintain cell electrophysiology [22].

Even though bone cells are non-excitable cells, stress-generated piezoelectricity has been shown to stimulate bone precursor cell proliferation and differentiation to restore the injured site, making electroactive materials and electrical stimulation a valid tool for bone regeneration strategies [156].

Stem cells are also sensitive to electrical cues and their differentiation can be modulated by electrical stimulation and culture on electroactive materials. NSC ability to undergo neuronal and glial differentiation is boosted by electroactive material and exogenous electrical field [157,158]. The use of electrically conductive material have also been shown to promote the neuronal differentiation of adult stem cells derived from non-neural tissue without the addition of neuron-specific growth factors and cytokines [159,160]. Cardiomyocyte differentiation of iPSCs, ESCs, and MSCs is possible with the use of chemically defined media, but it dramatically increases if coupled with electroactive materials, showing protein expression, cell morphology, and contractility of the natural tissue [161,162]. Similarly, MSC differentiation into osteoblasts can be achieved with specific osteogenic media; but it is further supported by the aid of electroactive scaffolds [163].

### 3.3. The Importance of the “Nanoscale”

**Figure 8:**
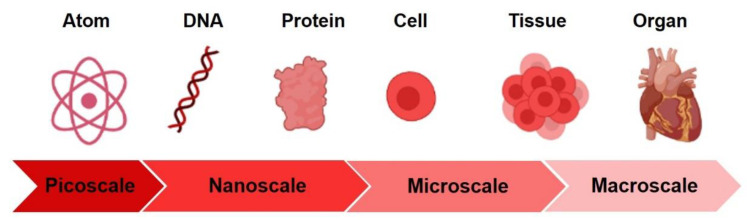
The relative scale of biological molecules and structures.

Cells have micro and nanoscale sensitivity because the extracellular environment presents a variety of spatially defined cues in the sub-micron to micron scale (Figure 8).

At the nanometer level, the extracellular environment affects sub-cellular behaviors such as the organization of cell adhesion molecule receptors. At the micron level, the extracellular environment affects cellular and supracellular characteristics such as cell morphology and [163]. The nanoscale physical features of the scaffolds can affect cell behavior. Natural tissues have indeed a hierarchical structure ranging from the macroscale (>1 mm) to the microscale (1 µm–1 mm), and the nanoscale (<1 µm). As a result, individual cells (typically in the size range 10–50 µm) respond in different ways to structures at different length scales. It has been shown that integrin receptors possess characteristic dimensions on the order of 10 nm [164]. The basement membrane of organs consists of nanoscale fibers (line topography) and pores (holes) that range in diameter from a few nanometers to several hundred nanometers [165]. The tubular fibers of collagen also have nanoscale dimensions [166] and laminin shows a nanoscale texture as well [167]. Given that cell ECM is patterned down to the nanoscale, cell-biomaterial interactions in scaffolds can be optimized by incorporating features of nanoscale dimensions. Indeed, surfaces topographically structured at the submicron scale can affect a wide variety of growth parameters, such as cell adhesion, morphology, viability, genic regulation, apoptosis, motility, and differentiation [168]. Evidence from nanoscale topography analysis suggests that nanoscale features eliciting a cell response are in the same size range (50–70 nm) that is associated with integrin cluster formation [169]. Further studies showed that scaffold nanotopography can control cell fate by altering cell and nucleus shapes, hence activating intracellular signal transduction and silent gene expression [125,170]. This is particularly true for neurons that, thanks to their growth cones, sense and actively respond to the surface nanotopography with a surprising sensitivity to variations of few nanometers [171].

Molecular deposition and lithographic techniques allow the patterning of tissue-specific molecules with nanometer resolutions. For example, the deposition of molecules that promote and support neuronal adhesion, growth, and differentiation on regenerating scaffolds enables the selective adhesion and growth of neural cells and a controlled neurite extension along the geometric pattern [172]. Apart from peptide/protein nanopatterns, nanomaterials and nanotechnology tools can also be used to develop special scaffolds able to recapitulate the architecture of structural proteins within ECM and the nanoscale features that model native ECM nanotopography [142]. Nanomaterials take advantage of their unique molecular features to induce, with high specificity, a number of desired physiological responses in target cells and tissues, while minimizing undesirable effects [173]. The peculiar mechanical and chemical properties of nanomaterials can be exploited for integration with native tissue in long-term implants; moreover, their nanoscale features have the potential to interact with the biological system at the molecular scale, while offering elevated levels of control [174].

Combinations of stimulatory cues may be used to incorporate nanoscale topographical, biochemical, and electrical cues in the same scaffold to provide an environment for tissue regeneration that is superior to inert scaffolds. This approach—able to precisely regulate cell differentiation, morphology, and polarization—is fundamental for the development of next-generation scaffolds suitable for clinical applications.

### 3.4. Role of the Biomolecular Corona

Interactions between the surface chemistry of nanomaterials and surfaces of biological components (proteins, phospholipids, organelles, DNA etc.) are crucial to determine the effects on cells and tissues. As soon as a nanomaterial comes in contact with a biological fluid (i.e., cell culture media, blood or interstitial fluid) it is coated with ions and proteins and develop a new interface which is often referred to as the protein corona or biomolecular corona (BC). This layer at the nanobio interface defines the biological identity of the nanomaterial, determining cell interactions, uptake and clearance [175]. Protein adsorption by GBMs has been reported in numerous studies. Umadevi and Sastry [176] analyzed non-covalent interactions on the surface of carbon nanostructures and highlighted that the graphitic lattice of graphene allowed hydrophobic interactions and strong π-π stacking with aromatic amino acids (Phe, Tyr, Trp) with binding energies between 15 and 20 kcal mol^-1^. Surface chemistry is key in tuning the strength and type of interactions. Epoxide, hydroxyl and carboxyl groups on the surfaces favor hydrogen and electrostatic bonding with proteins, facilitating adsorption on GO compared to pristine graphene or RGO. GBMs have been shown to strongly bind to different serum proteins such as albumin, fibronectin (Fn), collagen, and laminin [177]. Therefore, when cells grow onto a graphene-based scaffold, they show an enhanced capacity to form focal adhesions by clustering integrin molecules and favoring cell adhesion [125]. In addition, GBMs capacity to adsorb proteins results in trapping growth factors produced by the cells during their differentiation. Such growth factors can progressively be released during cell maturation, allowing a continuous supply, which is suitable for long-term cell differentiation [177].

Not only graphene physical properties favor the adsorption of proteins, but they also offer tremendous opportunities for the covalent functionalization of protein active motifs and chemical groups [178]. Such approach allows the stable attachment of signalling molecules to the graphene structure to influence cell behaviour, but it also simplifies combination of graphene with both natural and synthetic polymers for the development of superior scaffolds combining multiple cues for cell growth and differentiation.

## 4. Nanotoxicology and Functionalization

### 4.1. In Vitro Cytotoxicity

The use of graphene-based nanomaterials (GBMs) does not come without possible concerns about in vitro cytotoxicity and in vivo biocompatibility. As anticipated in Section 3.4, the biomolecular corona (BC) plays an important role in regulating the fate and toxicity of nanomaterials that interface with a biological environment. Due to the unique and distinct physico-chemical properties of graphene and its derivatives, there is an enormous variability at the nano-bio interface which leads to different intrinsic toxicological effects. Moreover, nanomaterials are often pre-bound to chemical moieties that originate from the manufacturing process, from stabilizers used in their preparation or from exposure to gasses or buffers, all of which might further impact biocompatibility. Therefore, any generalization would be inaccurate, possibly misleading and must be avoided [25,179].

Pristine GBMs have been shown to have a dose- and time-dependent in vitro toxicity in both procaryotic [180,181,182,183] and eucaryotic cells [184,185,186,187]. Graphene has a hydrophobic nature that often causes irreversible aggregation in cell culture media and it has been reported to agglomerate on cell membranes causing physical damage [188]. Conversely, oxidized derivatives of graphene, such as graphene oxide (GO) and reduced graphene oxide (RGO), are more hydrophilic and show little aggregation in biological buffers resulting in lesser cytotoxicity [179]. According to Chatterjee and co-workers [189], who performed a comprehensive study about biological interaction of oxidized graphene derivatives, GO and RGO had similar toxic responses with different dose-dependency and distinct molecular mechanisms which were attributed to their peculiar surface oxidation status. However, the presence of oxidative functional groups on the surface can lead to the generation of reactive oxygen species (ROS). In addition, if they are not correctly washed, graphene nanomaterials might retain residual chemicals applied to separate the graphitic layers or during the fabrication of oxidized derivatives.

To solve these problems, novel green approaches for nanoparticle synthesis and modification have been developed, involving the use of biocompatible surfactants and reducing agents. According to Askari et al. [190], graphene nanosheets can be synthetized in the presence of Herceptin, a natural antibody, using an ultrasonic-assisted exfoliation method. The toxicity of graphene was tested in 3D spheroid cultures of human breast adenocarcinoma cell line (SKBR-3) to better mimic the natural tissue micro-environment. The authors concluded that that presence of Herceptin and its residues on graphene nanoparticles created a biocompatible platform suitable for cell growth. In another study, Narayanan et al. [191] described a facile and green synthesis of reduced graphene oxide by the deoxygenation of GO under aqueous alkaline conditions in the presence of soluble starch as a reducing agent (SRGO). The cytotoxicity of SRGO on skin fibroblasts was evaluated using a Wst-1 assay and showed that SRGO showed a substantial increase in cell viability at high concentrations (200 µg mL^-1^) compared to non-reduced GO. The authors also investigated the hemocompatibility profiles of the nanomaterials and revealed that both caused a hemolysis effects compared to negative controls. However, SRGO did not exhibit a direct proportionality between hemolytic activity and concentration, with hemolysis staying as low as ~4.9% in maximum concentration samples.

### 4.2. Hemocompatibility and Interaction with Immune System Cells

Understanding interactions between nanomaterials and blood is key to determining in vivo biocompatibility due to the unavoidable contact between the two. Thanks to the protein corona effect, nanoparticles that touch blood or enter the bloodstream are coated by a milieu of proteins that may undergo conformational changes, exposing new epitopes and promoting phagocytosis or elimination from the circulation [192]. Nanomaterials can cause hemolysis and activate or interfere with clotting and coagulation cascades [193], seriously hindering the health of the organism.

The hemolytic property of nanoparticles is influenced by their distribution size, shape, surface charge and chemical composition [194]. Jaworski et al. [195] studied pristine graphene, RGO and GO effects on chicken embryo red blood cells (RBCs) and reported altered RBC morphology with loss of biconcavity. All of the nanomaterials exhibited dose-dependent hemolytic activity towards RBCs, with highest hemolysis rates observed at 5 mg ml^-1^. Pristine graphene showed the highest hemolysis (73%), followed by RGO (42%) and GO (27%), correlating with the degree of surface oxidation. Lower hemolytic concentrations and activity have been reported by other groups [193]. However, according to Duan et al. [196] the hemolytic potential of GO can be largely reduced by pre-incubating it with BSA or FBS, exploiting their extremely high protein adsorption ability. In another work, Sasidharan et al. [197] provided evidence that pristine graphene and GO have excellent hemocompatibility showing no hemolysis, platelet activation or plasma coagulation up to a relatively high concentration (75 μg mL^−1^) and under in vitro conditions. The authors also highlighted that pristine graphene had the potential to upregulate the production under sterile conditions of pro-inflammatory cytokines, such as IL-6 and IL-8. Cytokines are soluble glycoproteins released during an inflammatory response that recruit immune cells in order to tackle foreign bodies that have entered the organism.

Understanding interactions of GBMs with the immune system is of considerable relevance both from a toxicological and biomedical perspective. The BC of carbon-based nanomaterials is abundant in complement proteins. These proteins play a central role in modulating the immune and inflammatory responses towards intruders and may be a key factor in generating chronic ailments (such as allergy and sterile inflammation) by recruiting neutrophils and macrophages [198,199]. In addition, complement activation can promote cell-mediated immunity by enhancing generation of antigen-specific immunoglobulins by B-cells, activation of T-cells and uptake by dendritic cells [200]. Neutrophils and macrophages are part of the reticuloendothelial system (RES) which is responsible for the uptake and clearance of foreign bodies that have entered the organisms: the former are normally the first to intervene in an inflammation reaction, whereas the latter arrive later and promote tissue healing. It has been reported that macrophages better uptake hydrophilic systems compared to hydrophobic graphene since it is poorly dispersible in water and remains blocked on the cell surface [197]. Similarly, neutrophils are involved in nanoparticle clearance and it has been shown that exposure to carbon-based nanomaterials may upregulate neutrophils infiltration in tissues [201]. Carbon nanomaterials are also known to trigger apoptosis and/or cell death in macrophages, causing significant impairment in the immune resistance of subjects if used in vivo. However, Lin et al. [202] reported in a recent study that macrophage viability and activation are found to be mainly unaffected by few-layered graphene (FLG) at doses up to 50 µg mL^−1^ and therefore it is of little toxicity for M1 and M2 human macrophages, even though it triggers cell stress, ROS and inflammatory cytokines. Notably, neutrophils and macrophages are cleared from the circulation via the liver, spleen and kidneys and there is evidence that bone marrow may also play a major role in their clearance [203]. Therefore, nanomaterials carried by these cells can accumulate in those districts, causing unexpected issues and altering their fate.

### 4.3. In Vivo Biocompatibility

In vivo biodistribution and pharmacokinetics of GBMs have been studied in small and large animal models [204,205,206,207,208,209] in order to investigate the adsorption, distribution, metabolism and excretion (ADME). The fate of nanomaterials in organisms is influenced not only by their properties, but also from the pathway of exposure. Thanks to the wide range of potential applications of GBMs in biomedicine, exposure can occur in a number of ways including inhalation, intratracheal instillation, oral gauge, injection (intraperitoneal, intravenous or subcutaneous) or through debris generated from worn or biodegraded implants [204,210]. Once inside the organism, nanomaterials can make their way into the bloodstream even if not directly injected there and spread throughout the body. In addition, there is evidence that GBMs can diffuse across biological barriers such as the blood-air, blood–brain, blood-testis or blood-placental barrier, and accumulate in organs causing acute and chronic inflammation, tissue lesions and necrosis [211,212]. Krajnak and coworkers [213] examined graphene nanoparticles of different sizes and different forms (carbon black, graphene, GO and RGO) to determine if pulmonary exposure resulted in changes in vascular function and expression of acute response markers in mice. It was observed that while graphene altered gene expression in cardiovascular system, no changes were produced in the peripheral vascular function. On the other hand, pulmonary exposure to the oxidized forms of graphene had a more acute effect on heart and kidneys and repeated exposure might lead to injury or dysfunctions. Another study reported that GO provokes severe and persistent injury in mice lungs including granulomas persisting for up to 90 days [214]. Biodistribution experiments on intratracheally instilled carbon-14 labeled FLG showed that even if it was mainly retained in lungs, it was also redistributed to the liver and spleen passing through the air-blood barrier. However, no detectable absorption of FLG was observed when administered orally [212]. Conversely, radioactive-labelled RGO given through an oral gauge was rapidly absorbed in the intestine, metabolized by the kidneys and then excreted via urine [215]. Intravenous injection of GO in mice elicited blood platelets aggregation and extensive pulmonary thromboembolism, while low uptake was observed in the RES [216]. Surprisingly, a recent study by Newman et al. [217] highlighted that GO sheets accumulate preferentially in the spleen and progressively biodegrade over nine months. They evaluated the potential consequences of this prolonged accumulation and found limited effects on spleen histopathology and splenic function. Cell-mediated immune response was measured by determining the populations of T lymphocytes, specifically CD4+ and CD8+ cells as the major immune component of the splenic white pulp. Moderate changes were seen in both cell populations in mice injected with GO (2.5, 5, and 10 mg/kg) at both 24 h and one month after administration and no significant differences in the levels of the proinflammatory cytokines IL-6 and TNF-α were detected at any time point compared to control. However, they registered a significant drop in anti-inflammatory cytokines expression at 24 h and at the one-month time point for all tested GO doses. The authors concluded that reduction in cytokine expression after GO treatment may indicate the involvement of the innate immune system in regulating the effects of GO.

### 4.4. Minimizing GBM Toxicity

Although the inherent toxicity of graphene and its derivatives is a major drawback for their biomedical applications, it is a well-known problem, and different strategies have been developed to overcome it. In an attempt to enhance their overall safety and minimize the risks for adverse reactions in humans from exposure, Bussy et al. [218] offered a set of rules for the development of graphene and its derivatives: (1) use small, individual graphene sheets which are more efficiently internalized by macrophages in the body and removed from the site of deposition; (2) minimize aggregation using hydrophilic, stable, colloidal dispersions of graphene sheets; (3) use excretable graphene material or chemically-modified graphene that can be degraded effectively. Biological responses to these nanomaterials depend on various properties and it has been reported that smaller particles and higher oxidation improve biocompatibility [219]. Most importantly, variations in surface chemistry play a major role determining their toxicity and pharmacokinetic profile [220]. Highly hydrophobic graphene tends to aggregate in aqueous solvents thanks to intermolecular attractive Van der Waals forces, π-π stacking, hydrogen bonds and electrostatic interactions [221]. This tendency makes it hard to manipulate and characterize their biocompatibility and it has been suggested that the high percentage of controversial results in toxicity statistics could be owing to the dissimilarities in GBMs solubility [222]. Therefore, improving the dispersion of graphene-based nanomaterials in various solvents is a prerequisite for their further applications. Recent strategies include sonication, stabilization with surfactant and surface functionalization.

Wojtoniszak et al. [223] showed that GO and RGO exhibit a surfactant-dependent toxicity by comparing the homogeneity of GO and RGO dispersions in phosphate buffered saline (PBS) and cell viability on mice fibroblasts L929 cells. Three different dispersants were used, namely PEG, poly(ethylene glycol)-*block*-poly(propylene glycol-*block*-poly(ethylene glycol) (Pluronic P123), and sodium deoxycholate (DOC). The authors concluded that both materials had relatively good cytocompatibility in the 3.125–12.5 µg mL^−1^ range, with lowest toxicity detected in PEG-stabilized GO.

The BC can be tuned and modified by exploiting the ability of GBMs to adsorb moieties from the culture medium. As previously discussed, biological and bioactive species (DNA, carbohydrates and proteins) can be used as surfactants to stabilize graphene nanomaterials in aqueous solution, paving the way for different biomedical applications [191,224,225]. Pre-incubation in protein solutions was shown to form a thin coating on nanomaterials in suspension, minimizing cytotoxicity by limiting their direct interaction with cells. It has been reported that GO and RGO coated with BSA [226], FBS [227] or serum proteins [228] showed attenuate cytotoxicity and could improve biocompatibility. In addition, another interesting method of surface modification is functionalization through exposure to a specific enzyme, peptide or antibody [190,229]. Bussy et al. [230] exposed human lung carcinoma (A549) and bronchial epithelial (BEAS-2B) cell lines to GO and analyzed its effect in serum-free HEPES-buffered salt solution (BSS), Dulbecco’s phosphate-buffered saline (PBS), and the normal media recommended for these cell lines (F12 for A549 and RPMI for BEAS-2B). Surprisingly, they reported more pronounced cellular responses in both BSS and PBS, but not in F12 or RPMI, and concluded that the interaction between GO and cells may differ depending on the concentration of salts and ions present in the aqueous environment. These charged moieties could influence nanofiller aggregation, bundling, stacking, or other colloidal properties of the negative surface-charged nanoparticles. In summary, the presence of proteins and other moieties in the cell culture medium influences the results on cytotoxicity and we could consider that GO and RGO might not be hemolytic in vivo where an abundant BC forms on their surfaces and protects the nanomaterial. These types of non-covalent surface functionalization are not stable in prolonged circulation and it is important to consider the dynamic changes of the BC as the nanoparticles translocate from one biological compartment to another or from the ECM intracellular locations [231]. 

Covalent functionalization is another strategy for enhancing solubility in different matrices and is frequently used to obtain nanocomposites, as previously discussed in Section 2. However, covalently bonding molecules to the surface leads to the disruption of the graphitic lattice changing its electronic and transport properties [232]. A number of surface modifications allow to obtain more hydrophilic GBMs with remarkably reduced toxicity. According to Kiew and coworkers [233], graphene-based nanomaterials with hydrophilic surfaces weaken the opsonin–protein interaction and could avoid being recognized by macrophage, thus an inflammatory response. Among the different strategies, the combination with polymeric materials represents a commonly used approach to overcome the limitations of graphene-based nanomaterials in biomedicine [234]. Several studies have reported that covalent modification with polyethilenglycole (PEGylation) can reduce cytotoxicity resulting in increased biocompatibility and stability in physiological buffers [235]. PEG is known to prolong particle circulation in the blood due to its ability to camouflage particle surfaces, sterically shielding against opsonization and uptake by the RES cells [192]. Other approaches involve the covalent attachment of conductive polymers (such as poly(pyrrole), poly(aniline), poly(allylamine)) or biodegradable synthetic polymers (e.g. poly(lactic) acid, poly(glycolic) acid, poly(lactide-co-glycolide)) or natural polysaccharides such as chitosan [236], alginate, hyaluronic acid and dextran (DEX). Like PEGylation, dextran coating reduces the adsorption of proteins the surface and improves biocompatibility. Compared with non-functionalized GO, GO–DEX conjugates showed improved stability in physiological solutions, accumulation in liver and spleen after intravenous injection, and most importantly clearance from body within a week without causing noticeable short-term toxicity [237].

Finally, graphene and its derivatives have been used in combination with biocompatible polymeric matrices to obtain conductive nanocomposites with enhanced cell adhesion, differentiation and biocompatibility [238]. These materials trigger reduced biological responses without the impairment of the GBMs capability to cross cell membranes and deliver therapeutic species [239]. As our lab has recently shown, organic-functionalized carbon nanofillers dispersed in a polymeric poly(L-lactic) acid exhibited enhanced cell viability (~90%) and supported cell growth [59], while having interest effects on the differentiation of neuronal precursors [50] and human circulating multipotent stem cells [47,159].

## 5. Examples of Tissue Regeneration

### 5.1. Bone Regeneration

Bones possess a remarkable regenerative capacity, as they maintain the ability to remodel themselves throughout adult life and they can repair fractures spontaneously [240]. After bone damage, soluble factors accumulate at the injury site and recruit mesenchymal stem cells, which, in turn, proliferate and differentiate toward osteoblasts. Subsequent calcification of the region results in a woven bone, which is finally remodeled by the renewing and resorptive actions of osteoblasts and osteoclasts [241]. Despite this regenerative process, there are instances where injuries may require clinical intervention to be completely healed. Autologous bone graft is a standard medical procedure for the treatment of bone-related diseases. Unfortunately, it is mostly limited by the availability of appropriate donor tissue [242]. Several scaffolds have been developed to enhance bone regeneration to overcome this issue and some representative examples are reviewed in this section.

For instance, graphene was used to coat three-dimensional hydroxyapatite scaffolds to support the growth and osteogenic differentiation of hMSCs. Scaffolds were found to be self-standing, as hMSCs differentiation did not require common differentiative molecules (i.e., dexamethasone or the bone morphogenetic protein 2) [160]. Moreover, graphene oxide was covalently linked to chitosan (CS), an animal-derived polymer already known to support cell adhesion and proliferation. The resulting polymer had better elastic modulus and hardness, which resulted in an increase in cell adhesion, spreading, proliferation, and formation of the extracellular matrix. Most importantly, cells grown onto GO-CS scaffolds showed an enhancement in calcium and phosphate deposition levels, a hallmark for osteoblastic differentiation [243].

Arnold and co-workers managed to enhance hMSCs osteogenic differentiation by directly functionalizing GO. Expressly, they set up an elegant universal synthetic procedure to covalently tether polyphosphates onto GO, generating a new phosphate-graphene material (CaPG) [244]. Their approach allowed them to obtain scaffolds with hydroxyapatite-like functionality at the interface, loaded with osteoinductive ions. They developed a 3D scaffold and assessed that its mechanical properties were comparable with bones (Young’s modulus up to 1.8 GPa, compressive storage modulus up to 291 MPa, shear storage modulus up to 545 MPa, and ultimate compressive strengths up to 300 MPa). When hMSCs were seeded onto those scaffolds, a significant increase in the osteogenic marker alkaline phosphatase (ALP) and increased calcium deposits were observed, even when cells were cultured in growth medium (designed to maintain multipotency). Histological analyses of mice tissue after scaffold implantation showed no apparent damage, toxicological effects, or inflammation up to 8 weeks after treatment. More importantly, CaPG scaffolds enhanced donor cells’ retention and provided differentiative signals favoring bone regeneration without using growth factors to direct osteogenesis.

Li and co-workers employed graphene oxide and lysozyme films to favor bone regeneration while minimizing the possibility of infection. Precisely by depositing overlapping layers of GO and lysozyme onto a chitosan base they obtained a construct not only able to support dental pulp stem cell growth and differentiation but also with improved antimicrobial activity. While GO is responsible for scaffolds stiffness and roughness, lysozyme improves the antimicrobial activity of GO by degrading the bacterial cell wall [245].

Among 3D scaffolds, Li and co-workers [246] provided an interesting proof of concept of the usage of 3D-printed alginate hydrogels as scaffolds for bone engineering. They used 3D-bioprinting to obtain gelatin-alginate scaffolds with defined porosity, then coated them with RGO. Although hydrogels are much less stiff than other composites, the authors observed a significant increase in adipose-derived stem cell (ADSC) differentiation toward the osteogenic lineage, as proven by the increase in ALP expression and calcification of the substrate.

Graphene oxide osteogenic potential was further investigated by Wu and co-workers, which grafted it with a peptide derived from the bone morphogenetic protein 2 [247]. GO-BMP2 was then bonded to silk-fibroin electrospun fibers to obtain biocompatible scaffolds that favor MSC adhesion and differentiation in vitro and in vivo and is are able to repair mice bone defects in less than 14 days.

### 5.2. Muscle Regeneration

Skeletal muscles made up most of the mass of the human body and are essential for motion and support. They are composed of multinucleated myofibers, which developed from mononucleated stem cell precursors during embryonic development. Satellite cells are unipotent stem cells that remain associated with adult myofibers and are responsible for muscle growth and regeneration [248,249]. Because of them, muscle tissue is endowed with a remarkable regenerative capacity, and most injuries sustained during everyday life fully recover via well-characterized processes [250]. However, severe injuries such as volumetric muscle loss and neuromuscular degenerative diseases, or aging, can result in significant muscular impairment, severely dampening life quality. In recent years, the possibility to produce scaffold recapitulating features of adult muscle tissue to enhance regeneration has drawn much attention, and several features that can enhance muscle regeneration have been identified. Among those, Gilbert and co-workers found substrate elasticity to be pivotal for muscle regeneration, as substrates mimicking tissue elasticity (~12 kPa) were able to sustain muscle stem cell self-renewal in vitro and differentiation in vivo [251]. Moreover, it was found that electroconductive scaffolds can enhance myoblasts fusion into myotubes in vitro, possibly by mimicking neuromuscular activity [252,253]. Starting from the observation that scaffold elastic properties are pivotal to resist the dynamic condition of the muscle tissue environment, Jo and co-workers [254] developed polyurethane/graphene oxide nanocomposite fibrous scaffolds to form a flexible and myogenic stimulating matrix for tissue engineering. They found nanocomposite to have better tensile strength, hydrophilicity, and biocompatibility than pristine materials. When they seeded mouse skeletal muscle cells C2C12 (a standard model for muscle differentiation studies) onto their scaffolds, they found an enhancement in cell adhesion and spreading, as demonstrated by the increase in the expression of actin and vinculin. Scaffolds were also capable of inducing muscle differentiation, as immunocytochemistry against myosin heavy chain (MHC, a marker for mature muscle cells) and RT-PCR against MyoG, α-actinin, and MyoD (markers for differentiating muscle cells) showed an increase directly proportional to GO concentration. Most importantly, they also found that scaffolds were able to sustain dynamic tensional stimuli, which, in turn, further increased the expression of differentiative markers. 

As muscle cells are aligned along the fiber axis, materials patterned with surface features resembling native extracellular environment can influence mechanotransduction and favor cell differentiation. Park and co-workers [255] employed femtosecond laser ablation (FLA) to produce GO and RGO-based micropatterned conductive PAAm-hydrogels, which can support muscle differentiation in vitro and proved to have good stability in vivo. All scaffolds resembled muscle tissue Young’s modulus, but only rGO-based ones possessed enough conductivity to deliver signals to cells. FLA allowed them to pattern scaffolds with 20 µm wide, 10 µm deep canals, and only scaffolds with a pattern distance comparable to cell dimension (50–80 µm) proved to affect differentiation. Specifically, when fusion index (i.e., the ratio of nuclei inside myotubes to all nuclei) and nuclear shape (which becomes less rounded during differentiation) were considered, it was found they could be improved by 50 and 80 µm patterned scaffolds independently on their conductivity. Morphological analyses were confirmed by immunocytochemical and qRT-PCR analyses, which demonstrated an increase in the expression of differentiative and mature myoblast markers (i.e., MHC, MyoG, and MyoD). In spite of this, conductivity proved to be pivotal to enhance cell aspect ratio, and electrical stimulation (2V, 10ms duration, 1 Hz) enhanced myotube formation with respect to untreated control. Hydrogels were also found to be suitable for implantation, as they remained intact for 4 weeks after subcutaneous implantation in mice, proving they can be a good platform for tissue implantation. 

Besides skeletal muscle, cardiac muscle regeneration has drawn much attention because of the severity of heart diseases. Cardiomyocytes are specialized muscle cells which have a crucial role in the propagation of electric signal throughout the heart. Unlike skeletal muscle cells, cardiomyocytes have a reduced regenerative potential, and, after damage, are often replaced by scar tissue, which may lead to pathological heart failure. In an elegant comparative study, Lee and co-workers [256] compared the effects of gelatin methacrylic (GelMA) functionalized with either CNTs, GO, or RGO on the structural organization and functionality of rat primary cardiomyocytes. Even though all scaffolds resembled the elastic modulus of the heart, GO functionalized scaffolds exhibited low conductivity. Moreover, GO and RGO functionalized scaffolds displayed higher surface roughness compared to the GelMA and CNT-GelMA ones. Despite those differences, all scaffolds proved to support cell attachment and proliferation; however, they had different effects on cell differentiation. Specifically, when cells were stained against Cx43 (indicating electrical and metabolic coupling between cells), troponin-I and sarcomeric α-actin (both involved in muscle contraction), where enhanced only on RGO and CNT-GelMA but not in GO-GelMa. Moreover, even RGO failed to enhance the expression of troponin-I. Cells were further analyzed by patch-clamp to determine the extent and shape of the membrane action potential. Based on results, they found that CNT-GelMA led to the formation of ventricular like cardiomyocytes, whereas GO-GelMA resulted in an atrial-like phenotype. Instead, RGO-GelMA led to cells with a mixed phenotype. This finding suggests that different properties of the graphene derivative in the scaffold can be exploited to fine-tune cardiomyocyte phenotype.

In the context of injectable gels, Choe et al. developed an RGO-modified alginate gel and studied its antioxidant activity for cardiac tissue repair post myocardial infarction (MI) [257]. One of the hallmarks of MI is the high oxidative stress of heart tissues due to the formation of reactive oxygen species. Mesenchymal stem cell transplantation is a promising treatment for repairing heart tissues post MI, but after transplantation, their survival is compromise by the oxidative stress of the tissue. In their study, Choe et al. encapsulated hMSC in alginate microgels with a spherical shape (235 ± 11 μm diameter) suitable for easy injection. Nanocomposite microgels displayed higher cell viability than GO- and RGO-free beads. To further improve survival, hMSCs were first enclosed in GO/alginate hydrogels and then GO was reduced. Nanocomposite microgels showed greater scavenging activity in all assays, while the graphene-free counterpart had a negligible antioxidant activity. These injectable anti-oxidizing nanomaterial-embedded microgels were able to scavenge radicals and lower the oxidative stress post MI, support MSC viability and maturation, thus increasing therapeutic activities and regeneration of infarcted tissues.

### 5.3. Nerve Regeneration

The nervous system represents the most intricate and vulnerable system in the human body, as, despite its pivotal importance, it is substantially unable to regenerate itself after injury. Because of its vital role, its organization is extraordinarily complex. Briefly, the nervous system comprises two main classes of cells: the glial cells and neurons. Neurons act as functional units, as they are characterized by peculiar electrophysiological features which allow them to rapidly transmit information between each other. Connections are established during neuritogenesis by the sprouting of dendrites and axons from the cell body. Specifically, each growing axon is tipped by the growth cone, a complex molecular machinery that senses environmental stimuli to guide growth toward the proper target [258]. On the other hand, glial cells consist of various specialized cell types (including Schwann cells, oligodendrocytes, and astrocytes) that regulate homeostasis, form myelin sheets around axons, and provide support and protection for neurons by maintaining a proper microenvironment [259]. Anatomically, the nervous system has been divided into the central (CNS) and peripheral (PNS) nervous system. Besides their different physiological role, they also respond differently to damages. Central nervous system regeneration is made more challenging, mainly because adult CNS is naturally resilient to cell repair and differentiation. For instance, after axotomy, glial cells of the CNS secrete inhibitory cues and form a physical and chemical barrier, the glial scar, which prevents regenerating axons to cross the injury site and reach their new target. Moreover, the basal expression of anti-regenerative cues such as chondroitin sulfate proteoglycans, Nogo-A, and myelin-associated glycoproteins, semaphorin 4D, and ephrin, is upregulated, further suppressing the capacity of the axonal growth cone to elongate [260,261,262]. Conversely, PNS neurons are endowed with a higher regenerative capacity due to the lack of CNS inhibitory factors [263]. 

Because of the inability of central neurons to regenerate, traumatic brain injury, and spinal cord injury have profound adverse effects on life quality and are a significant cause of mortality [264]. Efforts from the scientific community to address this issue resulted in several pharmacological and surgical therapeutic strategies [265]. However, in recent years graphene and its derivatives emerged as intriguing tools to design biomaterials mimicking tissue properties, encapsulate biomolecules and favor stem cell differentiation or tissue regeneration [266,267,268,269]. Recently, Quian and co-workers [266] used 3D printing and layer-by-layer casting methods to produce graphene and polycaprolactone scaffolds, which improved axonal regrowth and remyelination. Their technique allowed them to optimize quality control, mechanical strength, drug delivery distribution, and achieve the ideal electric conductivity for nerve growth. To increase scaffold biocompatibility, they coated it with polydopamine (PDA) and arginylglycylaspartic acid (RGD), which can encapsulate small molecules and favor cell adhesion, respectively. When tested with rat-immortalized Schwann cells, they found the optimal proliferation and viability rates on scaffolds at 1% graphene in PCL and that those scaffolds were able to support cell proliferation or up to seven days. Moreover, they found a higher expression of vinculin and *N*-cadherin on PDA/RGD-G/PCL scaffolds rather than on control scaffolds, indicating that graphene can have a role in promoting cell adhesion. Western blotting and qRT-PCR analyses indicated that not only scaffolds were able to induce expression of neural markers (such as glial fibrillary acidic protein, Class III ß-tubulin, and S100) but also they increase the expression of neurotrophic factors (NGF, BDNF, GDNF, and CNTF), which are vital to establishing a permissive environment for nerve regeneration. Moreover, when Schwann cell-loaded PDA/RGD-G/PCL scaffolds were grafted onto Sprague Dawley rats, histological and immunohistochemical observations 18 weeks after surgery suggested that regenerated nerves were well organized, lacked scar tissue and, most importantly, functional recovery was comparable to autograft implants.

As neurons require network formation to acquire proper function, tools to build 3D neuronal networks are required to enhance their function. An elegant method to encapsulate neurons onto a self-assembled micro-roll made of a bilayer of graphene and parylene-C [268], provided a proof of concept for designing a 3D neuronal network, which might also serve as a platform for modeling neurodegenerative diseases or producing cells suitable for transplantation. Their approach allowed them to create a support that allows neurons to interact with their surroundings without mixing with the external population, thus keeping a precisely controlled cell distribution. They exploited a sacrificial layer of calcium alginate to support a graphene layer, which was then coated with a parylene-C layer. Finally, the bilayer was patterned with an array of microscale pores to allow axons, but not cell bodies, to contact surrounding cells. Self-assembly into a tubular structure was induced by treating the sandwich with ethylenediaminetetraacetic acid (EDTA) to de-polymerize the alginate layer. Accessibility of reagents to the internal of the micro-roll was assessed by Ca^2+^ imaging in response to the addition of glutamate: encapsulated hippocampal neurons showed a coherent and coordinated response, and no delay with the response of external neurons was observed. Moreover, the formation of functional synapses between neurons was demonstrated both by immunocytochemistry against synapsin I, which is expressed by neurons at the synapse puncta, and by monitoring the synchronization of spontaneous Ca^2+^ waves. Besides serving as support for cell growth, the authors claim the graphene in their scaffold might serve as an electrode for electrophysiological recording and neuronal activity stimulation.

**Figure 9:**
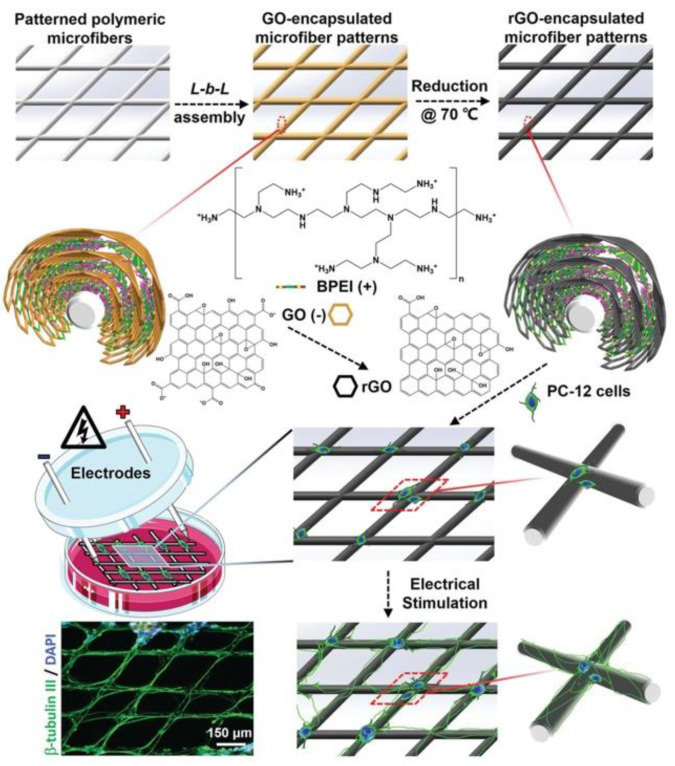
A diagram of the key procedures toward neuronal-like network formation with the guidance of conductive microfiber patterns under electrical stimulation [272].

In order to study the role of substrate conductivity in neuronal network formation and alignment, Wang and colleagues developed a 3D conductive GO-coated scaffold based on printed PLCL microfibers using a near-field electrostatic printing (NFEP) [270]. NFEP is a technique that combines electrostatic spinning and 3D printing that allows to obtain fiber sizes of a few micrometers and complex architectures [271]. By manipulating the motion of the collection surface along X-Y-Z axes, NEFP easily generates arbitrary patterns (2D or 3D). PLCL scaffolds with different fiber overlay angles, diameters, and spatial organization were coated with GO, which was then reduced to RGO in situ without damaging the architecture. Depending on the layer thickness, RGO coating improved electrical conductivity while increasing surface roughness. The scaffolds were then used to assess the correlation between electrical stimulation (ES) and neurite outgrowth of the pheochromocytoma-derived PC12 cell line and primary neurons from hippocampal tissue of embryonic mice. ES enhanced neurite outgrowth and alignment with respect to control without ES stimulation. Strikingly, while neurite outgrowth resulted in being strictly correlated with the strength of the electric field, its directionality did not seem to influence neurite alignment. However, it was found that neurite outgrowth tightly followed the orientation of the smaller microfiber pattern and a more dispersive distribution of neurites was observed on fibers with higher diameters, where neurites had a higher tendency to branch out and lose their directional orientation (Figure 9).

Glial cells are as necessary as neurons to ensure proper nervous system functionality, therefore their regeneration after injury is as crucial as neuronal restoration [272]. Specifically, oligodendrocytes are responsible for myelination of central neurons and must be restored to ensure proper neuronal connectivity. The most common way to obtain oligodendrocytes is to differentiate multipotent NSCs or induced pluripotent stem cells (iPSCs). However, the process has proven to be challenging, as it requires long culture periods (up to 150 days) and has a limited yield [273]. Shah and co-workers [267] developed a nanocomposite PCL-GO scaffold, which allowed for NSCs differentiation into oligodendrocytes in just 6 days of culture. They treated electrospun PCL nanofibers of 200–300 nm diameter with oxygen plasma to render their surface hydrophilic, then GO was deposited on their surface at either 0.1, 0.5, or 1 mg/mL. Finally, laminin, an ECM protein essential for adhesion, growth, and differentiation of NSCs, was used to coat scaffolds. Rat hippocampal NSCs displayed significant differences in cell morphology after just 6 days of culture. Moreover, concentrations of GO as low as 0.1 mg/mL were able to strongly enhance the expression of the myelin basic protein (MBP), a marker specific for oligodendrocyte differentiation. The absence of an effect on the expression of the neuronal marker Tubβ3 and the astrocytic marker GFAP further suggested those scaffolds were able to selectively direct differentiation toward the oligodendrocytic lineage. When they treated cells grown on PCL-GO with integrin signaling inhibitors, they observed a steep decrease in oligodendrocyte markers. This observation suggested that the GO-coating of the nanofiber scaffolds might promote differentiation through specific microenvironmental interactions that activate integrin-related intracellular signaling. 

Besides rigid scaffolds, biocompatible conductive hydrogels have attracted much attention because of their ability to better reproduce the mechanical properties of host tissues. Javadi and co-workers [269] developed a biocompatible hydrogel, based on polyurethane (PU), poly(3,4-ethylenedioxythiophene) (PEDOT) doped with poly(4-styrenesulfonate) (PSS) and liquid crystal graphene oxide (LCGO). They obtained a formulation with excellent conductivity, tensile modulus, and yield strength to support neuronal stem cells differentiation toward neurons and glial cells (as proven by the increase of the neuronal marker Tubβ-3 and the astrocyte marker GFAP). The authors claim LCGO liquid crystal nature synergistically combined with the properties of PEDOT:PSS to increase hydrogel mechanical and electrical properties.

Starting from evidence that the cholinergic system is involved in several neuron protective processes, cortical plasticity, and functional recovery after brain injury, Pradhan and co-workers developed a choline-graphene oxide functionalized (CFGO) injectable hydrogel based on poly(acrylic acid). Not only their hydrogels were able to support neuronal cell growth and differentiation, but they also stabilized the actin cytoskeleton. As choline is involved in enhancing neural recovery in TBI treatment, they injected their hydrogels in mice with parietal cortex brain injuries. They found scaffolds were able to restore cortical loss in just 7 days of treatment [274].

### 5.4. Wound Healing

GBMs have also been employed as fillers for wound healing hydrogels. Rehman and co-workers [275] developed RGO-GelMA hydrogels which enhance migration of fibroblasts, keratinocytes and endothelial cells in vitro and favor angiogenesis, in vivo, in chicken embryos. The authors speculate that this property could be due to an increase in intracellular ROS levels caused by RGO. In another study, Li and co-workers [276] developed N-acetyl cysteine (NAC) loaded GO-collagen membranes. In this formulation, GO has been reported to enhance mechanical properties and water retention of the collagen scaffolds, whereas NAC is used to lower ROS levels in the damaged tissue. The membrane accelerated cell migration, maturation and angiogenesis, leading to rapid skin regeneration. Moreover, the expression of profibrotic factors was found to be downregulated, indicating those scaffolds could promote scarless wound healing. A common problem of wound healing hydrogel is their vulnerability to bacterial infection [277]. To solve this problem, Yan and co-workers [278] developed an Ag reduced GO sodium alginate film which not only is able to inhibit bacterial growth but also to stimulate rapid wound healing in vivo.

## 6. Conclusions

Graphene-based scaffolds have been proven to be versatile tools in mediating tissue regeneration, as highlighted by the examples of in vitro and in vivo applications that have been discussed in this review. However, much more effort is required from the scientific community to clarify and rationalize their mechanism of action. It is clear that different composites can be employed to obtain similar results and yet subtle changes in scaffold formulation may result in completely different results. Therefore, a systematic analysis of the effects of scaffold composition on differentiation is required in order to disentangle the role of each scaffold component on cell fate. This would allow for a direct comparison between different scaffolds and a finer rational design. Moreover, it needs to be stressed that most applications rely on qRT-PCR data to prove successful differentiation. However, this approach reveals only an average trend in cell differentiation, without detecting potentially significant and biologically important differences between cells seeded onto different spots of the scaffold. Even when immunocytochemical data are provided, most authors fail to mention whether or not those data are representative of the whole sample or are just isolate cases. Coupling scaffold engineering with single cell RNA sequencing would overcome those limitations, allowing both a better understanding of scaffold effects on cell physiology and comparisons between the in vitro culture and the in vivo reference.

To date, scaffold engineering has focused on regeneration of a single tissue. However, clinical applications often require grafting of whole organs. Obtaining a scaffold that is able to efficiently reproduce a whole organ, or even multiple tissues (e.g., innervate muscles), has proven challenging and further studies are required before any viable clinical usage. In our opinion, finding the rationale behind graphene regulation of cell fates will allow us to obtain scaffolds that can reliably support and differentiate cells in a number of pre-determined types at the same time.

Although a molecular rationale for graphene-mediated effects is still lacking, it is remarkable that graphene-based scaffolds are able to determine cell fate more rapidly and efficiently than any other differentiation protocol, even without the addition of exogenous pro-differentiation factors. Indeed, in recent years research on mechanotransduction has unveiled several details on how nanotopography and stiffness stimuli are perceived and transduced by cells, whereas further efforts are needed to elucidate the contribution of other chemical and physical stimuli from the tissue environment. In particular, conductivity is of special interest to neuronal cell fate and differentiation, as it is specific to the nervous system. In addition to the aforementioned nanotopographic features, graphene and GBMs are endowed with tunable conductivity. Therefore, graphene-based nanomaterials represent a useful and cost-effective tool to enhance neuronal differentiation and tissue repair.

## Data Availability

Not applicable.
